# Integrated Electrochemical Aptamer Biosensing and Colorimetric pH Monitoring via Hydrogel Microneedle Assays for Assessing Antibiotic Treatment

**DOI:** 10.1002/advs.202309027

**Published:** 2024-09-09

**Authors:** Fatemeh Keyvani, Peyman GhavamiNejad, Mahmoud Ayman Saleh, Mohammad Soltani, Yusheng Zhao, Sadegh Sadeghzadeh, Arash Shakeri, Pierre Chelle, Hanjia Zheng, Fasih A. Rahman, Sarah Mahshid, Joe Quadrilatero, Praveen P. N. Rao, Andrea Edginton, Mahla Poudineh

**Affiliations:** ^1^ Department of Electrical and Computer Engineering Faculty of Engineering University of Waterloo Waterloo Ontario N2L 3G1 Canada; ^2^ Department of Bioengineering McGill University 815 Sherbrooke St. W Montreal Quebec H3A 0C3 Canada; ^3^ School of Pharmacy University of Waterloo Waterloo Ontario N2L 3G1 Canada; ^4^ Department of Kinesiology and Health Sciences University of Waterloo Waterloo Ontario N2L 3G1 Canada

**Keywords:** aptamer, microneedle, pH sensing, therapeutic drug monitoring, wearable sensing

## Abstract

Current methods for therapeutic drug monitoring (TDM) have a long turnaround time as they involve collecting patients' blood samples followed by transferring the samples to medical laboratories where sample processing and analysis are performed. To enable real‐time and minimally invasive TDM, a microneedle (MN) biosensor to monitor the levels of two important antibiotics, vancomycin (VAN) and gentamicin (GEN) is developed. The MN biosensor is composed of a hydrogel MN (HMN), and an aptamer‐functionalized flexible (Flex) electrode, named HMN‐Flex. The HMN extracts dermal interstitial fluid (ISF) and transfers it to the Flex electrode where sensing of the target antibiotics happens. The HMN‐Flex performance is validated ex vivo using skin models as well as in vivo in live rat animal models. Data is leveraged from the HMN‐Flex system to construct pharmacokinetic profiles for VAN and GEN and compare these profiles with conventional blood‐based measurements. Additionally, to track pH and monitor patient's response during antibiotic treatment, an HMN is developed that employs a colorimetric method to detect changes in the pH, named HMN‐pH assay, whose performance has been validated both in vitro and in vivo. Further, multiplexed antibiotic and pH detection is achieved by simultaneously employing the HMN‐pH and HMN‐Flex on live animals.

## Graphical abstract

This work presents a multiplexed assay based on biocompatible hydrogel microneedle biosensors. These assays enable simultaneously detecting therapeutic drugs as well as pH in a minimally invasive manner.

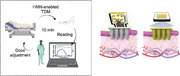

## 1 Introduction

The therapeutic window (the effective drug concentration with minimal toxicity) of many drugs; specifically, antibiotics, is narrow, making their precision dosing crucial.^[^ ^1^, ^2^ ^]^ If the level of these antibiotics falls below the minimum effective concentration, this can lead to dangerous outcomes (i.e., death due to sepsis). Conversely, concentrations above the window may result in permanent hearing loss and nephrotoxicity due to toxicity.^[^ ^3^, ^4^, ^5^ ^]^ Patients receiving narrow therapeutic window antibiotics usually undergo therapeutic drug monitoring (TDM), where the drug's serum concentrations are measured, and its dosage is adjusted accordingly.^[^ ^2^ ^]^ Severe infection (i.e., sepsis) can also cause metabolic acidosis which leads to a series of downstream effects, harming hemodynamic stability, and increasing the risk of mortality.^[^ ^6^ ^]^ Therefore, in addition to TDM, monitoring the pH level during infection and its evolution over the course of antibiotic treatment is beneficial for tracking treatment effectiveness.^[^ ^6^, ^7^, ^8^ ^]^

**Figure 1:**
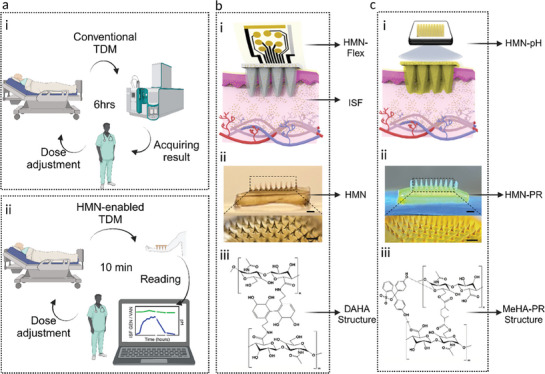
Schematic illustrating the conventional a‐i) and HMN‐enabled a‐ii) TDM and pH monitoring. Schematic illustrating the HMN‐Flex assay employed for VAN/GEN monitoring b‐i) which is composed of an HMN b‐ii) and a flexible electrode, the chemical structure of the DAHA‐based HMN is shown (b‐iii). Schematic showing the HMN‐pH assay for pH sensing c‐i), which employs the HMN‐PR c‐ii), the HMN‐PR is composed of MeHA hydrogel and PR, and its chemical structure is illustrated c‐iii). Scale bars = 1 mm. The schematics were created using BioRender and CorelDraw Graphics, and chemical structures were drawn by ChemDraw.

Current methods of TDM include invasive blood draws followed by time‐consuming, laborious, and costly blood processing and analysis (mainly via immunoassays and liquid chromatography) to determine the level of antibiotics in the bloodstream,^[^ ^2^ ^]^ and their turnaround times can be upward of 24 hours (h)^[^ ^9^ ^]^(**Figure** **1**a,i). Further, due to the complex, costly, and lengthy processes involved in these conventional methods, TDM is usually only performed at a specific time(s) postadministration, which may not provide the optimum temporal resolution required for different antibiotics.^[^ ^10^ ^]^ The conventional TDM also cannot capture ongoing fluctuations in drug levels and cannot be used to draw an accurate picture of individual pharmacokinetic (PK) characteristics and how the body interacts with the drug.^[^ ^2^, ^9^, ^10^ ^]^ In addition, blood sampling is painful and can be challenging for children, critically ill, and elderly patients.^[^ ^11^ ^]^

The shortcomings of conventional TDM methods raise the need for technologies to perform TDM in a minimally invasive and continuous manner with improved temporal resolutions (Figure 1a‐ii). Recently, a new generation of biosensors has emerged that can continuously measure biomolecules in vivo using an electrochemical biosensor based on sensitive and specific structure‐switching aptamer probes.^[^ ^12^, ^13^, ^14^, ^15^, ^16^ ^]^ When bound to its target, the aptamer undergoes a reversible conformational change, producing a change in faradaic current that can be measured using standard voltammetry techniques, enabling continuous tracking of rising and falling concentrations of the target analytes in real‐time.^[^ ^14^, ^15^, ^16^ ^]^ The aptamer‐based electrochemical detection has been applied for continuous measurement of different drugs such as vancomycin (VAN) and gentamicin (GEN), two key antibiotics, in whole blood.^[^ ^15^, ^16^, ^17^ ^]^ However, the complicated and invasive design (i.e., insertion into the jugular vein which requires surgery) and/or the short monitoring capacity (a few hours) are the main shortcomings. In addition, the presence of blood cells and large protein molecules can cause electrode fouling, which reduces signal transduction and limits continuous in vivo detection.^[^ ^15^, ^17^, ^18^ ^]^

Monitoring therapeutic drugs in interstitial fluid (ISF) can address these challenges, as ISF comprises lower concentrations of cells and large molecules; thus, the fouling effect is minimized.^[^ ^19^, ^20^ ^]^ The composition and pH of ISF have also been found to closely resemble that of blood, mainly due to the exchange of small molecules (like albumin, CO_2_, and phosphates) between ISF and plasma.^[^ ^21^, ^22^ ^]^ Further, ISF can be accessed using microneedles (MN) in a minimally invasive manner.^[^ ^19^ ^]^ Solid microneedle biosensors have been recently reported to access ISF and perform TDM in transdermal ISF.^[^ ^12^, ^13^ ^]^ For instance, MN electrodes fabricated via micromachining and sputtering techniques were developed for detecting irinotecan and doxorubicin, two commonly used chemotherapeutic drugs.^[^ ^13^ ^]^ In another work, 3D‐printed solid MNs were fabricated for continuous monitoring of tobramycin antibiotics both in vitro and in live animal models.^[^ ^13^ ^]^ In these studies, upon insertion of solid MNs into the skin, the sensing elements come into immediate contact with the dermal ISF, potentially damaging the aptamer probes attached to solid MNs, and adversely impacting the sensor's functionality. Additionally, the stiffness of the reported solid MNs limits their suitability for use on soft and curved skin, leading to progressive retraction of the MNs and diminished signal over time.^[^ ^12^, ^23^ ^]^ To prevent signal retraction, the need for subcutaneous placement of magnetic plates via skin incision made the deployment of solid MNs invasive.^[^ ^23^ ^]^ Further, solid MNs made from materials such as metals, are biologically incompatible, and their application may trigger immune responses or tissue reactions when inserted into the skin. Needle breakage within the skin is also another critical concern.^[^ ^24^, ^25^ ^]^ In the event of breakage, the retrieval of the fragmented needles can be a challenging and invasive process.^[^ ^25^, ^26^ ^]^ A summary of recent electrochemical aptamer‐based sensors developed for TDM is presented in Table S1 (Supporting Information).

To address these challenges, we developed a hyaluronic acid (HA)‐based hydrogel microneedle (HMN) integrated with flexible (Flex) electrodes (HMN‐Flex) and employed it for the detection of VAN and GEN (Figure 1b). In our system, HMN arrays serve as a matrix for extracting the ISF; along with the antibiotics, while Flex electrodes immobilized with specific aptamer probes measure VAN/GEN concentrations. We studied the performance of the HMN‐Flex system for detecting VAN and GEN within their therapeutic ranges using ex vivo skin models. We also showed that the HMN‐Flex can effectively detect VAN and GEN in live animal models, which are healthy rats injected with different dosages of VAN/GEN antibiotics. The ISF PK measurement using our system correlates with blood measurement. Further, given the importance of pH monitoring during bacterial infection, we developed another HA‐based HMN system for pH measurement that can be used to assess the success of antibiotic treatment (Figure 1c). In this colorimetric pH sensing assay, HA‐based hydrogel was combined with phenol red (PR); a pH‐sensitive molecule, and formed a pH‐responsive HMN that we named HMN‐pH assay (Figure 1c‐i). The HMN was used for ISF extraction and the change in PR color under varying pH levels was employed to report the pH status in the clinical ranges of 7–8. The performance of the colorimetric HMN‐pH assay was tested on ex vivo skin models as well as in vivo models. The color changes were translated to the real pH values using a simple RGB method captured by a smartphone camera. Additionally, we tested the performance of our HMN assays for simultaneous and multiplexed detection of GEN and pH in vivo. These results show that our system can be employed for effective TDM and pH measurement in both standard and critical care settings, potentially revolutionizing conventional TDM.

## 2 Results and Discussion

### 2.1 The Hydrogel Microneedle‐Flexible Electrode (HMN‐Flex) and Hydrogel Microneedle‐pH (HMN‐pH) Sensing Strategy

**Figure 2:**
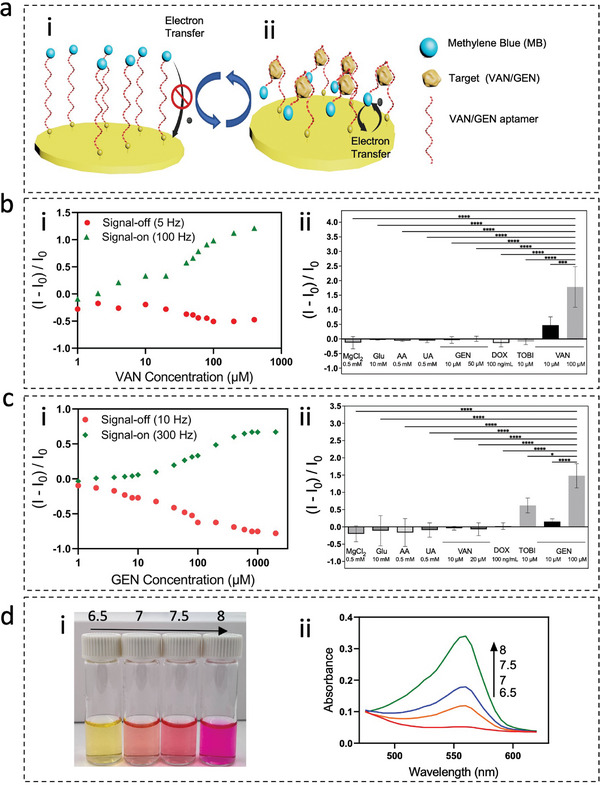
Characterizing the performance of Flex electrodes for detecting VAN/GEN and the PR solution for measuring pH. Schematic illustrating the aptamer‐based electrochemical detection of VAN/GEN a), slow electron transfer happening in the absence of target a‐i), while faster electron transfer happening when the aptamer is bound to target a‐ii). Characterization of signal‐on and signal‐off frequencies for Flex electrode immobilized with VAN aptamer b‐i), and the specificity test for VAN with statistical analysis b‐ii). Characterization of signal‐on and signal‐off frequencies for Flex electrode‐immobilized with GEN aptamer c‐i), and the specificity test for GEN with statistical analysis c‐ii). The number of individual replicates (*n*) = 3, the error bar represents the standard deviation (STD), and the following abbreviations have been used: MgCl_2_, Magnesium Chloride; Glu, Glucose; AA, Ascorbic Acid; UA, Uric Acid; DOX, Doxorubicin; TOBI, Tobramycin. Image representing the change in PR color in response to different pH d‐i), and their correspondence absorbance measurement d‐ii). To assess whether the difference between non‐specific targets and the specific targets for VAN and GEN are significant, we have performed a one‐way ANOVA and post hoc Tukey tests. The result for one‐way ANOVA (*p* = 0.05) was found significant (*p* < 0.0001), and the post hoc Tukey test also presented significant differences in the response to the specific targets compared to the non‐specific ones where the significances are shown in GP style (0.1234 (ns), 0.0332 (^*^), 0.0021 (^**^), 0.0002 (^***^), *p* < 0.0001 (^****^)). The schematics were created using CorelDraw Graphics.

HMN‐Flex assay consists of a dopamine‐based hyaluronic acid (DAHA) HMN for ISF extraction and Flex electrodes that are functionalized with specific aptamers for target detection (Figure 1b‐i). DAHA polymer was synthesized based on our previously reported protocols^[^ ^27^, ^28^ ^]^ and the DA conjugation was confirmed using ^1^H nuclear magnetic resonance (NMR) and ^13^C NMR (Figures S1 and S2, Supporting Information). To make a chemically crosslinked patch, the pH of the DAHA solution was increased leading to the covalent linkage of the carbon atoms of benzene rings and forming the DA‐DA conjugate sites (Figure 1b‐ii,iii). The electrodes for antibiotic detection were fabricated by following a standard Flex electrode micro/nanofabrication protocol, using polyimide as the substrate (Figure S3, Supporting Information). The Flex electrode is a conventional three‐electrode system with a silver/silver chloride (Ag/AgCl) reference electrode and gold (Au) counter and working electrodes (WE). The WE surface was functionalized with the previously reported VAN or GEN aptamer probes^[^ ^16^, ^18^ ^]^ whose one end was conjugated with thiol and the other end was labeled with methylene blue (MB), a redox reporter (**Figure** **2**a). The thiol group enables aptamer immobilization on the gold surface through gold‐thiol self‐assembled chemistry.^[^ ^29^ ^]^ The MB redox reporter signals the presence of the target of interest.^[^ ^15^, ^17^, ^30^ ^]^ Upon insertion into the skin, the HMN swells, and the VAN and the GEN, present in ISF, diffuse into the swelled network of hydrogel and are transported to the Flex electrode surface. In the absence of VAN or GEN, the MB redox reporter is distanced from the electrode surface; thus, the electron transfer happens slowly, and a lower current is observed (Figure 2a‐i). Once the aptamers recognize and bind to their respective targets, they undergo a conformational change that brings the MB‐modified terminus of the aptamer closer to the surface of the electrode, leading to a faster electron transfer and producing a higher electrochemical response (Figure 2a‐ii).

To monitor pH during TDM, we synthesized a methacrylated hyaluronic acid (MeHA) polymer by modifying HA with methacrylic anhydride (MAA) which acts as the polymer backbone of the HMN‐pH patch. To generate a crosslinked HMN patch for pH detection, photoinitiator (PI), and the crosslinking agent, N,N′‐ methylenebisacrylamide (MBA), were mixed with MeHA‐PR complex and applied to the polydimethylsiloxane (PDMS) mold.^[^ ^31^, ^32^ ^]^ Upon drying, the patches were exposed to UV to form a crosslinked patch via covalent attachment of MeHA carbon−carbon double bonds with the crosslinking agent under the presence of PI (Figure 1c‐ii,iii). The chemical structure and degree of methacrylation were confirmed by performing ^1^H NMR and ^13^C NMR (Figures S4 and S5, Supporting Information). The PR was entrapped within the MeHA network due to the multiple hydrogen bonding formation between HA functional groups and PR's hydroxyphenyl (Figure 1c‐iii). Following this process, we were able to fabricate HMN‐pH patches that are sensitive to pH changes while their sharp needles enable effective skin penetration. We should note that DAHA was not used as the polymer backbone of the HMN‐pH patch, because of its inherent yellow/brown color.

### 2.2 Characterizing the Performance of Flexible (Flex) Electrodes for Vancomycin (VAN) and Gentamicin (GEN) Sensing and Phenol Red (PR) Solution for pH Measurement

Continuous electrochemical measurement in complex biofluids like ISF is faced with two major challenges; i) the individual sensor's response to the target might vary due to the variations in sensor fabrication, and ii) the electrode is prone to fouling, reducing the sensor's performance.^[^ ^14^, ^15^, ^17^, ^30^, ^33^ ^]^ To account for the sensor‐to‐sensor variations and prevent signal drift over time, we deployed the previously reported “dual‐frequency” approach^[^ ^17^ ^]^ where the ratio of the electrode's response at two different frequencies; a signal‐on and a signal‐off frequency; is measured. Signal‐on frequency refers to the frequency at which the current increases by the increase in target, and signal‐off refers to the frequency at which the signal decreases by an increase in target concentration.^[^ ^15^, ^17^, ^18^, ^30^, ^34^ ^]^ Normalizing the signal obtained from the signal‐on frequency to the one obtained from the signal‐off frequency would compensate for the sensor‐to‐sensor variability and the sensor drift.^[^ ^17^, ^33^ ^]^

The signal‐on and signal‐off frequencies are unique to each aptamer and should be calculated within a system. Therefore, we first determined the signal‐on and signal‐off for VAN and GEN aptamers immobilized on the Flex electrodes. Buffer solutions were spiked with different concentrations of VAN or GEN while the electrochemical square wave voltammetry (SWV) technique was employed to interrogate the sensors at different frequencies.

The resulting current “I” was recorded, and the sensor's response was calculated via (I – I_0_) / I_0_, where “I” is the current at each condition and “I_0_” is the current in the blank buffer solution. The 5 Herts (Hz) and 100 Hz were found to be the signal‐off and signal‐on frequencies of the VAN sensor, respectively (Figure 2b‐i). The specificity of the VAN sensor was also examined by subjecting the VAN aptamer‐modified Flex electrode to different non‐specific targets commonly present in ISF, another antibiotic, tobramycin (TOBI), and the common cancer treatment drug doxorubicin (DOX). As shown in Figure 2b‐ii, the VAN sensor did not exhibit any response in buffers containing non‐specific targets.

For the GEN sensor, SWV measurements at 10 and 300 Hz frequencies showed signal‐off and signal‐on behaviors, respectively (Figure 2c‐i). Figure 2c‐ii demonstrates the response of the GEN sensor to non‐specific targets. Given that GEN and TOBI are both aminoglycosides with similar structures, a similar aptamer has been reported for their detection.^[^ ^16^ ^]^ This explains why our data shows an increase in current when the GEN aptamer is subjected to TOBI. The GEN sensor did not produce a response when exposed to DOX or other interferents. Additionally, employing the “dual‐frequency” method reduced the batch‐to‐batch variability and improved the sensor response (Figure S6, Supporting Information).

Next, we confirmed the applicability of PR for reporting changes in pH through absorbance measurement (Figure 2d). PR is a commonly used indicator of biological pH changes with an excellent color transition from yellow to pink/purple in the pH range of 6.5–8^[^ ^35^ ^]^ (Figure 2d,i). Our data also confirmed such behavior by measuring the absorbance rate at 560 nm of PR in different pH buffer solutions, suggesting PR as a good candidate for incorporation into HMN to achieve pH sensing (Figure 2d‐ii).

### 2.3 Hydrogel Microneedle (HMN) Patch Fabrication and Characterization

We next fabricated and characterized the DAHA‐HMN and MeHA‐HMN patches for skin penetration and ISF extraction. An important characteristic of our system that distinguishes it from previously reported TDM assays is the use of hydrogel‐based microneedles. HMNs are superior to other types of MNs as they can quickly and efficiently extract ISF, have excellent biocompatibility, are cost‐effective to manufacture, and have a high production yield.^[^ ^36^, ^37^, ^38^, ^39^, ^40^ ^]^ The most significant advantage of HMNs is their ease of insertion and removal, which minimizes the risk of skin damage during clinical applications.^[^ ^31^, ^32^, ^36^, ^39^ ^]^ Additionally, HA, a key component of our HMNs, has anti‐fouling properties that reduce nonspecific protein adsorption.^[^ ^40^, ^41^, ^42^ ^]^ This feature makes the HMN‐Flex system suitable for long‐term and continuous measurement within ISF.

**Figure 3:**
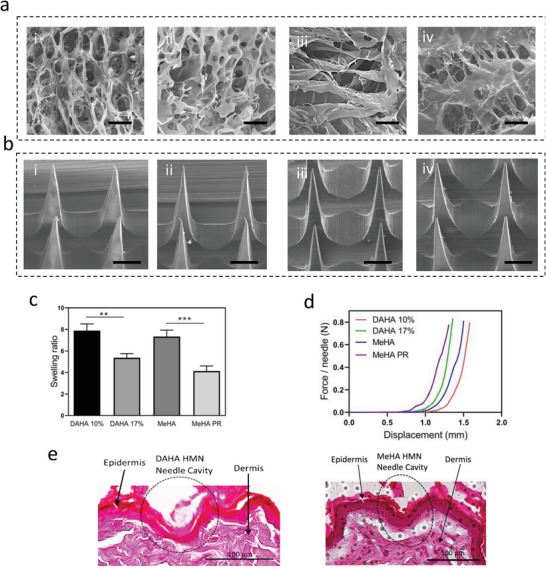
HMN Fabrication and characterization. SEM images representing the porosity of 10% DAHA hydrogel a‐i), 17% DAHA hydrogel a‐ii), MeHA hydrogel a‐iii), and MeHA‐PR hydrogel a‐iv), scale bar = 20 µm. SEM images showing the needle structure of the 10% DAHA HMN b‐i), 17% DAHA HMN b‐ii), MeHA HMN b‐iii), and MeHA‐PR HMN b‐iv), scale bar = 200 µm. The bar graph shows the swelling ratio of each type of HMN patches c), The error bar presents the standard error of the mean for three individual HMNs (*n* = 3), *p*‐values were determined using one‐way ANOVA, *p* < 0.05 (^*^), and *p* < 0.01 (^**^), and *p* < 0.001 (^***^). Graph showing the mechanical strength of the four types of HMN d). Histology images presenting the needle cavity for the 10% DAHA MN and MeHA‐PR (HMNs selected for fabricating the sensors) e) scale bar = 100 µm.

The ISF extraction by HMNs is influenced by their porous structure. Here we synthesized MeHA, MeHA‐PR, as well as DAHA with 10% and 17% DA and studied their porosity. **Figure** **3**a shows the scanning electron microscopy (SEM) images of the porous structure of the samples. The 17% DAHA had a more compact and less porous network than the 10% DAHA, owing to its higher degree of crosslinking (Figure 3a‐i,ii). The addition of the PR molecule also decreased the pore size of the MeHA hydrogel (Figure 3a‐iii,iv). Subsequently, HMN patches of MeHA, MeHA‐PR, 10% DAHA, and 17% DAHA with sharp needles were fabricated (Figure 3b). The HMN patches are 0.7 cm × 0.7 cm in size and have conical tips that are 850 µm tall (Figure 3b). To examine the swelling capability, the HMN patch's weight was measured before and after insertion into agarose hydrogel and the swelling ratio was calculated. The 17% DAHA and MeHA‐PR had lower swelling ratios when compared to 10% DAHA and MeHA, respectively (Figure 3c), an observation that aligns with the HMN's respective porosity levels. The 10% DAHA was employed in the HMN‐Flex assay because of its superior swelling behavior. To evaluate the mechanical strength of the HMN patches, dynamic mechanical analysis (DMA) was conducted to measure the force versus displacement of needles before their breakage. All HMN patches showed a mechanical strength greater than 0.4 N needle^−1^, which is necessary for successful skin insertion (Figure 3d).^[^ ^32^, ^39^ ^]^ We further assessed the skin penetration capability as well as the inflammatory response of HMN patches by applying them to the skin of live rats for 15 min. The skin was collected immediately after the patch removal and the surrounding tissue was assessed via hematoxylin and eosin (H&E) staining. Figure 3e illustrates that the HMN patches were able to penetrate the skin at a depth of 100 µm for DAHA and MeHA‐PR, with no apparent inflammation observed around the micropore. Our further experiments revealed that only a negligible (3%) release of PR from HMN‐pH patches can happen (Figure S7, Supporting Information).

### 2.4 Ex Vivo Characterization of HMN‐Flex and HMN‐pH Assays

**Figure 4:**
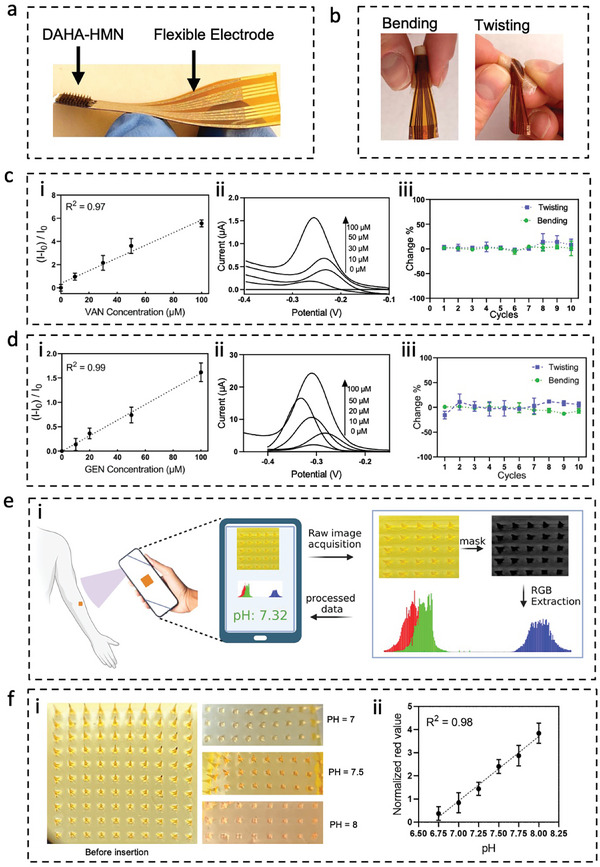
Ex vivo characterization of HMN‐Flex and HMN‐pH assays for antibiotic and pH sensing. Image representing the HMN‐Flex which is fabricated by integrating the DAHA‐HMN and flexible (Flex) electrode a). Image representing the bent and twisted states of HMN‐Flex b). The HMN‐Flex responses to different concentrations of VAN in buffer‐equilibrated porcine skin b‐i), and their respective SWV spectra at signal‐on frequency b‐ii), The percentage change in HMN‐Flex response after ten cycles of bending or twisting as compared to its response before bending or twisting (VAN concentration = 30 µm) b‐iii). The HMN‐Flex response to different concentrations of GEN in buffer‐equilibrated porcine skin c‐i), and their respective SWV spectra at signal‐on frequency c‐ii), the percentage change in HMN‐Flex response after ten cycles of bending or twisting as compared to its response before bending or twisting (GEN concentration = 20 µm) c‐iii). The “I” represents the dual‐frequency current (I signal‐on / I signal‐off) and “I_0_” shows the dual‐frequency current (I signal‐on / I signal‐off) in the absence of the target. The change % = 1 – (response after bending‐twisting / response before bending‐twisting) *100. The error bar represents the STD for at least three individual HMN‐Flex replicates (*n* ≥ 3). A schematic of the HMN‐pH working mechanism and the automated process of cropping, masking, RGB extraction, and pH evaluation d). Images of before and after insertion of HMN‐pH patches in porcine skin equilibrated with a buffer at biologically relevant pH (7–8) e‐i) and the corresponding calibration curve based on the change in red value of extracted RGB (of 3 individual replicates, with error bar showing the standard deviation) from the automated setup. e‐ii). The schematics were created using BioRender.

After ensuring the performance of the fabricated Flex sensor for accurately detecting VAN and GEN and characterizing the HMN patches, we proceeded with integrating the Flex electrodes with the HMNs to generate the HMN‐Flex (**Figure** **4**a) and assessed its performance using ex vivo porcine skin model (which resembles a medium similar to human skin).^[^ ^31^, ^32^, ^37^ ^]^

We also examined HMN‐Flex performance during bending and/or twisting cycles, the common movement imposed on the wearable sensors (Figure 4b). To detect different concentrations of VAN/GEN, porcine skins were equilibrated with buffer solutions containing varying concentrations of the target antibiotics overnight. Previously we have shown that the overnight incubation allows effective target diffusion into the skin.^[^ ^31^ ^]^ The next day, the skin pieces were dried from excess fluid, the HMN‐Flex (fabricated for specific detection of either VAN or GEN) was applied to the skins with different concentrations of the antibiotics of interest, and SWV scans at both signal‐on and signal‐off frequencies were obtained. The increase in the response obtained from the HMN‐Flex assay for VAN detection is found well‐correlated with the concentration of VAN in the porcine‐equilibrating buffer (R^2^ = 0.97) (Figure 4b‐i). Figure 4b‐ii displays an example of corresponding signal‐on SWV spectra for each tested concentration of VAN. The change in the HMN‐Flex response to 30 µm of VAN after ten consecutive bending and twisting cycles was also negligible, indicating the suitability of HMN‐Flex as a wearable VAN sensor (Figure 4b‐iii). Additionally, we applied the VAN HMN‐Flex on the equilibrated porcine skin and after 100 consecutive cycles of twisting and bending, the sensor response was measured in three different states: normal, bent, and twisted. No significant differences in the sensor response were observed in all three states (Figure S8a, Supporting Information), demonstrating the stable performance of the HMN‐Flex under different mechanical tensions.

Similar responses were observed for detecting GEN using HMN‐Flex assay. The increase in the HMN‐Flex response correlates well with the varying concentrations of GEN infused into the porcine skins (R^2^ = 0.99) (Figure 4c‐i). An example of corresponding signal‐on SWV spectra for HMN‐Flex is illustrated in Figure 4c‐ii. Similarly, the negligible changes in the HMN‐Flex response (to 20 µm GEN) after ten cycles of bending and twisting (Figure 4c‐iii) and under different mechanical tensions (Figure S8b, Supporting Information) show its suitability for wearable applications. It is noteworthy that the associated limit of detections (LOD) for VAN and GEN detection were 0.28 and 0.97 µm, respectively. Further, the HMN‐Flex was examined at VAN and GEN therapeutic ranges of 10–50 and 10–25 µm, respectively.^[^ ^4^, ^16^, ^43^, ^44^, ^45^, ^46^, ^47^, ^48^ ^]^

The performance of the HMN‐pH assay was evaluated using an accessible setup consisting of a smartphone camera and a 4X lens. After the removal of the HMN‐pH patch from the skin, an image was taken from the needle area. In this approach, the image goes through an automated process of cropping, masking, RGB (red, green, blue) extraction, and pH evaluation using MATLAB (Figure 4d). HMN‐pH patches were inserted into the porcine skin equilibrated with biologically relevant pH values from 6.75 to 8. After the removal of the patch, a color change similar to the buffer experiment was observed for each pH (Figure 4e). The RGB values of each patch were extracted using the foretold automated approach and a calibration curve was derived based on the change in red color (Figure 4e‐i,ii).

### 2.5 In Vivo pH and Antibiotic Detection Using HMN‐Flex and HMN‐pH Assays

Having demonstrated that HMN‐Flex and HMN‐pH could accurately detect VAN, GEN, and pH levels, we proceeded with testing their performance in vivo. Prior to the animal experiment, the biocompatibility of the hydrogels was tested via 3‐(4,5‐dimethylthiazolyl)−2,5‐diphenyl‐2H‐tetrazolium bromide (MTT) test,^[^ ^49^ ^]^ and retention in cell viabilities of 97% and 90% were obtained for DAHA and MeHA‐PR hydrogels, respectively (Figure S9, Supporting Information).

**Figure 5:**
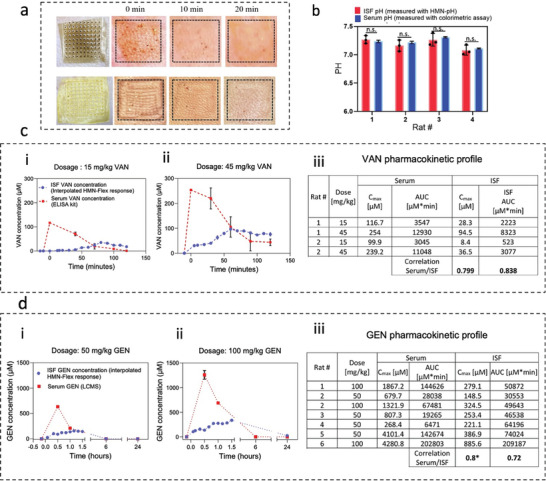
In vivo antibiotic and pH detection using HMN‐Flex and HMN‐pH assays. Photos representing HMN‐Flex (top row) and the HMN‐PR (bottom row) traces 0, 10, and 20 min after HMN removal a), Bar graph showing the ISF pH measured with the HMN‐PR (*n* = 3) next to the corresponding serum pH measured (*n* = 3) via conventional colorimetric methods in four individual rats b). The data is shown as mean ± standard deviation. *p*‐values were determined using one‐way ANOVA, *p* > 0.05 (n.s.). Graphs illustrating the ISF VAN concentrations (interpolated from HMN‐Flex response with an *n* ≥ 3 scans and error bar shows the standard deviation) before and after intravenous injection of 15 mg kg^−1^ c‐i) and 45 mg kg^−1^ along with serum VAN concentrations (measured with ELISA method with *n* = 3 and error bar shows the STD, where the not detected (ND) concentrations were reported as 0 and the values at the 0‐time point were drawn from extrapolating the other concentrations). ISF VAN concentrations for 15 mg kg^−1^ from left to right in µm is: 0 ± 0, 1.5 ± 0.46, 2.25 ± 0.06, 2.6 ± 0.51, 2.94 ± 3.02, 5.46 ± 3.30, 13.77 ± 1.26, 15.38 ± 3.10, 23.57 ± 1.9, 35.23 ± 2.04, 32.22 ± 2.14, 23.8 ± 0.93, 23.99 ± 4.30, 17.6 ± 4.4. Serum VAN concentrations for 15 mg kg^−1^ from left to right in µm is: 0 ± 0.1, 70.32 ± 7.6, 19.3 ± 11.15, 8.69 ± 2.34, 0.53 ± 0.25. ISF VAN concentrations for 45 mg kg^−1^ from left to right in µM is: 0 ± 0, 30.67 ± 5.51, 32.48 ± 3.66, 35.04 ± 2.8, 40.37 ± 3.87, 63.10 ± 7.40, 79.73 ± 4.18, 96.82 ± 1.6, 90.03 ± 2.73, 898.57 ± 2.93, 81.47 ± 1.95, 73.69 ± 0.28, 79.15 ± 2.67, 76.15 ± 6.94. Serum VAN concentrations for 45 mg kg^−1^ from left to right in µM is: 0 ± 0, 219.9 ± 41, 105.74 ± 40.8, 47.85 ± 38, 44.24 ± 13.23. The tabulated PK characteristics of C_max_ and AUC of two rats that received two different dosages of VAN c‐iii). Graphs illustrating the ISF GEN concentration (interpolated from HMN‐Flex response with an *n* ≥ 3 scans and error bar shows the standard deviation) in addition to serum GEN concentrations (measured with LCMS method with *n* = 3 and error bar shows the standard deviation, where ND was reported as 0 and the concentrations at 0‐time point were drawn from extrapolating the other concentrations) before and after intravenous injection of 50 mg kg^−1^ d‐i) and 100 mg kg^−1^ d‐ii) GEN. ISF GEN concentrations for 50 mg kg^−1^ dosage from left to right is: 0.37 ± 3.5, 25.5 ± 3.77, 101 ± 3.54, 89.7 ± 2.14, 121.06 ± 1.29, 137.9 ± 11.08, 155.4 ± 10.39, 149.9 ± 7.7, 144.4 ± 3.29, 0 ± 0, 0 ± 0. Serum GEN concentrations for 50 mg kg^−1^ from left to right is: 0 ± 0, 633 ± 14.13, 211.12 ± 1.7, 0 ± 0, 0 ± 0. ISF GEN concentrations for 100 mg kg^−1^ is 0.37 ± 1.45, 106.52 ± 15.22, 126.26 ± 9.54, 156.5 ± 10.6, 136.25 ± 17.26, 202.6 ± 15.11, 273.58 ± 9.35, 276.2 ± 8.4, 268.6 ± 6.7, 284.37 ± 8.57, 337.54 ± 27.77, 0 ± 0, 0 ± 0. Serum GEN concentrations for 100 mg kg^−1^ dosage from left to right is: 0 ± 0, 1260.14 ± 88, 688.65 ± 20, 0 ± 0, 0 ± 0). The tabulated PK characteristics of C_max_ and AUC of six rats that received two different dosages of GEN and the associated correlation coefficient values d‐iii).

To perform in vivo measurements, male Sprague Dawley rats were shaved, and either the HMN‐Flex or HMN‐PR was applied to the dorsal skin of the rats. The HMN‐Flex and HMN‐PR were applied on the rat skin for 2 h and 15 minutes (min), respectively (their maximum detection time duration) and the needle traces were observed right after patch removal (0 min) as well as 10‐ and 20‐min post‐removal (**Figure** **5**a). The results re‐confirm the effective needle penetration as well as the skin recovery upon patch removal.

The ability of HMN‐pH to monitor pH in vivo was tested on four healthy rats (Figure 5b). HMN‐pH patches were inserted in rats’ skin and were removed after 15 min, and the images were taken from the patch's needle area. At the same time, blood samples were collected from the rat tail. The pH of the patch acquired from blood serum was then analyzed using a colorimetric assay and was compared with the pH value from image analysis (Figure 5b). The HMN‐pH assay showed its accuracy and effectiveness for in vivo application by yielding measurements that had an average variance of less than 0.054 ± 0.09 pH compared to the measurements obtained from blood samples.

The HMN‐Flex for VAN or GEN detection was applied to the rat's dorsal skin and fixed using TaqMan tape. After 5 min, the sensor interrogation was started and continued until stable signals were achieved (typically happening after 10–15 min). At this point, the sensor response was recorded and documented as before the antibiotic injection (time 0). To examine the ability of HMN‐Flex to detect VAN or GEN in ISF, the rats were intravenously injected with two different dosages of antibiotics (15 mg kg^−1^ (mg kg^−1^) and 45 mg kg^−1^ for VAN and 50 mg kg^−1^ and 100 mg kg^−1^ for GEN).^[^ ^13^, ^19^, ^50^ ^]^ Since each rat could metabolize the drugs differently, the same rats were used to inject different dosages of VAN or GEN.^[^ ^51^ ^]^ The rats were used in two‐week intervals, ensuring that the administrated dosage was fully cleared from the rat's circulatory systems.^[^ ^13^ ^]^

Figure 5c‐i,ii represents the response of the sensor to two different VAN dosages. After the injection, the HMN‐Flex was interrogated every 5–10 min up to 120 min, and the resulting ISF concentration (interpolated from the recorded responses in Figure S10, Supporting Information) was reported (Figure 5c‐i,ii as well as Figure S11, Supporting Information). The venous blood samples were also collected every 30 min throughout the process and the serum samples were isolated for the following enzyme‐linked immunosorbent assay (ELISA) based VAN measurements (Figure 5c‐i,ii as well as Figure S12, Supporting Information). Previous studies have demonstrated that, following intravenous drug administration, serum drug levels exhibit an immediate decrease in blood concentration as they enter the elimination phase. In contrast, ISF drug levels initially undergo a distribution phase characterized by an increase in concentration, before entering the subsequent elimination phase, marked by a decrease in ISF concentration.^[^ ^50^, ^52^ ^]^ Our data reports the same behavior for VAN in serum and ISF. The serum VAN levels declined instantly after injection, while the response obtained from HMN‐Flex first increased after injection, followed by a decrease (Figure 5c‐i,ii). The response obtained from our HMN‐Flex sensor in the rats injected with 45 mg kg^−1^ of VAN was about three times higher than the rats received 15 mg kg^−1^ of VAN, indicating that HMN‐Flex can potentially differentiate between different dosages (Table S2, Supporting Information).

The estimation of PK parameters, such as the area under the time‐concentration level curve (AUC) and the peak concentrations (C_max_) was performed using two PK models describing the observed patterns of serum and ISF concentrations (Figure S13, Supporting Information) and the Spearman correlation test was used to assess the correlation between PK parameters in serum and ISF.^[^ ^53^ ^]^ The ISF VAN concentrations used for PK analysis were drawn by interpolating the sensor's response in the VAN calibration curve. The VAN AUC and C_max_ from ISF and serum were correlated with correlation values of 0.82 and 0.79, respectively (Figure 5c‐iii). This suggests that these parameters can potentially serve as tools for TDM in ISF and facilitate dose adjustments.^[^ ^1^, ^12^, ^13^, ^50^ ^]^

We also employed the HMN‐Flex assay for detecting GEN in ISF and drawing its PK profile. Figure 5d‐i,ii illustrates the ISF GEN concentration (interpolated from HMN‐Flex response using Figure S14, Supporting Information) before and after injecting the rats with 50 and 100 mg kg^−1^ of GEN. For this purpose, the HMN‐Flex response was monitored every 10 min up to 90 min, followed by two other measurements at 6‐ and 24‐hours (h) postinjection. We chose to measure GEN levels at the 6‐ and 24‐h time points based on previous research indicating that a more precise PK profile for ISF GEN can be obtained through extended monitoring.^[^ ^50^ ^]^ Rat's venous blood samples were collected throughout the measurements, and the obtained serum samples were analyzed via liquid chromatography‐mass spectrometry (LCMS) to correlate the sensor response with GEN serum levels (Figures S15–S17, Supporting Information). GEN serum levels decreased rapidly following injection (Figure 5d,i,ii), while, compared to blood, ISF GEN levels underwent a slower elimination (Figure 5d,i,ii). The concentration of ISF GEN was obtained by interpolating the HMN‐Flex response in the GEN calibration curve and used to draw the AUC and C_max_ of GEN for six individual rats (Figure S18, Supporting Information). Importantly, the AUC and C_max_ for ISF and serum GEN were found to correlate with correlation values of 0.72 and 0.8, respectively (Figure 5d‐iii). To the best of our knowledge, this is the first time reporting the changes in ISF GEN levels in the rat animal models. The lower concentration of VAN and GEN in the ISF, compared to blood, is aligned with prior research and can be explained by the high levels of VAN and GEN protein binding (50% and 30%, respectively), which in turn hinders the complete penetration of these drugs from blood to ISF.^[^ ^54^, ^55^ ^]^ It is noteworthy that the HMN‐Flex response did not increase when applied to rats injected with blank saline, further validating HMN‐Flex performance for specifically detecting VAN and/or GEN (Figure S19, Supporting Information). Importantly, the PK measurements obtained using HMN‐Flex closely matched those derived from conventional blood‐based measurements while the high temporal resolution offered by the HMN‐Flex assay can potentially revolutionize the current TDM.

### 2.6 In Vivo, Multiplexed pH and GEN Monitoring

**Figure 6:**
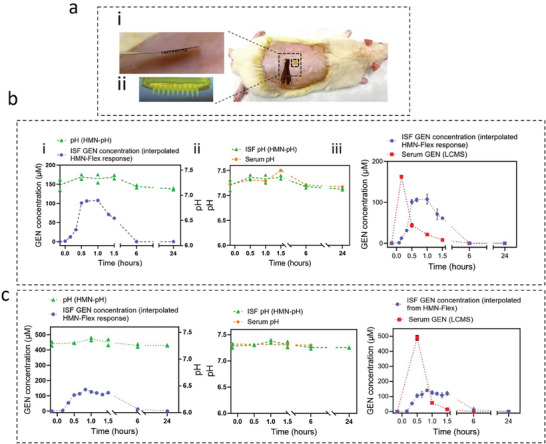
Multiplexed pH and GEN monitoring in vivo. Image representing the HMN‐Flex a‐i) and HMN‐pH a‐ii) applied on the dorsal skin of a rat for simultaneous GEN and pH monitoring. The HMN‐Flex response to fluctuating levels of GEN before and after injecting 50 mg kg^−1^ GEN (interpolated from HMN‐Flex response with an *n* ≥ 3 scans performed and error bars show the standard deviation (shown in b‐iii,c‐iii) as well as ISF pH values measured with the HMN‐pH (two individual patches per time‐point) in the first b‐i) and second c‐i) tested animal. The ISF pH next to the serum pH (measured with colorimetric assays where the values represent the mean of three individual measurements per time point and error bars showing the STD) at different time points throughout the experiments up to 24 h after GEN injection (50 mg kg^−1^) in the first b‐ii) and second c‐ii) tested animals. Serum pH in the first tested animal: 7.22 ± 0.006, 7.31 ± 0.007, 7.29 ± 0.001, 7.5 ± 0.006, 7.21 ± 0.012, 7.17 ± 0.008. Serum pH in the second tested animal: 7.43 ± 0.002, 7.17 ± 0.003, 7.38 ± 0.005. The ISF GEN concentration (from interpolated HMN‐Flex response) next to serum GEN levels (measured via the LCMS method, where *n* = 3 and the error bar represents the standard deviation) before and after GEN injection in the first b‐iii) and second c‐iii) tested animals. The first tested animal b‐iii), ISF GEN concentrations: 0 ± 0, 1.26 ± 1.79, 12.38 ± 1.54, 31.28 ± 2.66, 100.97 ± 5.56, 106.19 ± 4.60, 88.13 ± 4.70, 63.44 ± 1.95, 107.72 ± 12.12, 70.87 ± 13.09, 61.34 ± 0.65, 0 ± 0. Serum GEN concentrations: 0 ± 0, 162.43 ± 0.14, 43.72 ± 4.85, 21.43 ± 0.38, 8.37 ± 0.33, 0 ± 0, 0 ± 0. The second tested animal c‐iii), ISF GEN concentrations: 0 ± 0, 3.54 ± 3.79, 54.28 ± 11.17, 105.33 ± 12.94, 113.21 ± 20.57, 140.88 ± 6.01, 125.98 ± 13.52, 118.57 ± 10.98, 108.51 ± 18.25, 119.81 ± 12.62, 12.43 ± 15.76, 0.56 ± 0.99. Serum GEN concentrations: 0 ± 0, 489.16 ± 15.59, 57.68 ± 0.11, 14.35 ± 0.14, 0 ± 0, 0 ± 0. ND reported as 0.

The serum/ISF pH levels are disturbed during infection and can predict consequent complications and mortality.^[^ ^56^, ^57^, ^58^ ^]^ GEN is also a key antibiotic used in bacterial infection specifically in the pediatric population.^[^ ^59^, ^60^ ^]^ Therefore, simultaneous monitoring of pH and GEN levels in infected patients can provide great insight into the level and trajectory of infection and assist in maintaining appropriate GEN concentrations to combat infection and relieve downstream effects.^[^ ^7^, ^57^ ^]^ Here we applied the HMN‐pH and HMN‐Flex on the same rats to simultaneously monitor pH and GEN levels (**Figure** **6**a). The multiplexed pH and GEN measurements were performed on two different rats, shown in Figure 6b,c. The HMN‐Flex response was continuously monitored for 1.5 h followed by two other measurements at 6‐ and 24‐h postinjection using which the ISF GEN concentration was drawn and is shown as blue curves in Figure 6b,c. The green curves in Figure 6b,c also present the HMN‐pH response recorded before GEN injection and at 0.5, 1, 1.5, 6, and 24 h after injection. Employing both HMN assays on the rats has enabled concurrent measurements of the pH values and GEN levels in ISF (Figure 6b‐i,c‐i). The HMN‐pH response was validated by testing the pH level of serum samples collected at the same time points using the conventional colorimetric methods (Figure 6b‐ii,c‐ii). The level of GEN in the rat's serums was also measured via LCMS and used for validating the HMN‐Flex response and ISF GEN concentrations (Figure 6b‐iii,c‐iii). The GEN distribution and elimination pattern in serum and ISF are in line with the previously reported data (Figure 5). The accordance of serum GEN and pH levels with ISF GEN and pH levels validates the performance of the HMN‐Flex and HMN‐pH for detecting GEN and pH.

## 3 Conclusion

In the presented work, we developed two types of HMN biosensors; named HMN‐Flex and HMN‐pH, for the detection of antibiotics (VAN/GEN) and pH, respectively. The HMN‐Flex is composed of an HMN that extracts ISF and transfers it to the flexible electrodes. The flexible electrodes are functionalized with redox‐modified aptamers, which capture VAN/GEN and report their levels in ISF. Our data shows a correlation between the VAN/GEN PK profiles in ISF and blood, suggesting the application of HMN‐Flex for PK analysis as well as TDM, particularly effective for guiding drug dosage adjustments.^[^ ^61^ ^]^

To monitor the pH level in ISF, the pH‐responsive compound, PR, is incorporated into the HMNs leading to color changes in response to changes in pH. This color change is analyzed using an accessible and simple smartphone‐based and later quantified using an automated image‐extracting RGB to pH converter. We have also demonstrated that our HMN‐pH can be administered concurrently with our HMN‐Flex. This dual approach allows for the assessment of drug levels and pH, enabling the comprehensive monitoring of disease progression alongside TDM. We tested the performance of the HMN‐pH sensor in four individual rats. We examined the performance of the VAN HMN‐Flex sensor in two rats each receiving two different dosages of IV‐injected VAN, making a total of four individual experiments. We tested the HMN‐Flex for GEN detection in six rats, one of them received two different dosages, making a total of seven individual experiments. Two individual rats have also been used for the multiplexed VAN and pH testing.

The flexibility of both HMN patches and Flex electrodes make HMN‐Flex and HMN‐pH assays compatible with human skin. Since the sensing components do not come into direct contact with the skin, the risk of their damage or release is minimized. The HMN‐Flex is a universal platform, and the aptamers can be readily swapped to measure other types of drugs, in a continuous and minimally invasive manner. A breakthrough advancement in the current HMN‐Flex is to equip it with a closed‐loop drug delivery system. Such a system would sense the fluctuations in antibiotic levels in ISF and use those data to automatically adjust the drug dosage followed by delivering the adjusted dosage.^[^ ^62^ ^]^

The current techniques for TDM rely on blood samples, and further studies are necessary to establish ISF‐based TDM as a gold standard approach.^[^ ^50^, ^63^, ^64^ ^]^ Since antibiotics transfer from blood to ISF, changes in the ISF drug concentration occur with a delay. While this delay may restrict real‐time access to antibiotic levels in the blood, the ability to track drug fluctuations continuously offers significant advantages over existing methods, which typically provide PK information only every 6 h under optimal conditions.^[^ ^63^, ^65^ ^]^ Additionally, in the future, new PK models based on ISF drug levels can be developed that replace blood measurements. The HMN‐Flex sensor can aid in the creation of these new PK models.^[^ ^19^ ^]^

Similar to other ISF‐based sensors, the HMN‐pH sensor is limited by the lack of extensive data comparing ISF pH to blood pH. Additionally, future works should validate the performance of HMN‐Flex and HMN‐pH sensors in relevant infectious animal models. Prior to the clinical translation, the skin penetration of our HMN patches should be evaluated in pig models, as their skin closely resembles human skin. Using pig models with larger skin areas also allows for the application of multiple HMN sensors, facilitating the testing of our sensors' reproducibility.

## 4 Experimental Section

### Material

All of the DNA oligonucleotides were obtained from Integrated DNA Technologies (Coralville, IA, USA), and their sequences are listed in Table S1 (Supporting Information). The Pharma‐grade sodium Hyaluronic acid (HA, MW 300KDA) was purchased from Bloomage Co., Ltd (China). 10 × PBS, 2‐hydroxy‐4‐2‐hydroxy‐2methylpropiophenome (photoinitiator, PI), N′‐methylenebisacrylamide (MBA), methacrylic anhydride (MAA), D‐(+)‐glucose, uric acid (UA), sodium hydroxide, agarose (type I‐A, low EEO), serotonin hydrochloride powder, tris hydrochloride, N‐(3‐Dimethylaminopropyl)‐N′‐ethyl carbodiimide hydrochloride (EDC‐HCl), Dopamine Hydrochloride (DA), Phenol Red sodium salt, Propylene glyol methyl ether acetate (PGMEA or 1‐methoxy‐2‐propyl acetate), and other chemicals were purchased from Sigma Aldrich (Canada). Sodium chloride (CAS 7647‐14‐5 | 106404) was purchased from EMD Millipore. Calcium chloride (anhydrous), and magnesium chloride hexahydrate were purchased from Thermo Fisher. Potassium chloride was purchased from VWR Life Science. The porcine ear skin was obtained from a local supermarket. Tegaderm (Transparent Film Roll 16004) was purchased from 3 m. Polyimide sheets were purchased from DuPont Kapton HPP‐ST with a thickness of 200 µm. AZ4620 was purchased from AZ Electronic Materials Inc. Ma‐N 1410 photoresist and Ma‐D 533s photolithography developer was purchased from Microresist tech. GmbH. Titanium and gold and Chromium Thin films evaporated. SU‐8 2002 was purchased from KAYAKU advance materials. Chromium Etchant 1020, Gold Etchant TFA, and Hydrofluoric acid 49% were purchased from Tensene Company Inc.

### Hydrogel Synthesize

To fabricate 17%DAHA, first HA (2 g) was dissolved in 200 mL of Milli‐Q water for 12 h. EDC (485 mg) and NHS (291.5 mg) were then added to the HA solution. Next, dopamine hydrochloride (0.5 g) was added, and pH was set to five using hydrochloric acid and reacted for 24 h. The solution was then dialyzed at room temperature in dialysis tubing cellulose membrane (Sigma‐Aldrich, MWCO = 14 000) for 4 days at pH 4.5 to prevent dopamine auto‐polymerization. The solution was then freeze‐dried. DA conjugation was confirmed by ^1^H NMR to determine the successful DA conjugation. For synthesizing DA‐HA with a conjugation rate of 10%, the amount of EDC, NHS, and dopamine hydrochloride was reduced to half (EDC: 242.5 mg, NHS: 145.7 mg, DA: 0.25 g).^[^ ^27^, ^28^ ^]^

MeHA hydrogel was synthesized based on the modified protocol established before.^[^ ^32^ ^]^ Briefly, 2.0 g HA was dissolved in 100 mL Millipore water and stirred overnight under 4 degrees for complete dissolving. Subsequently, 1.6 mL MA was added to the HA solution followed by the stepwise addition of 3.6 mL of 5N NaOH solution, to adjust the pH to 8–9. The mixture was stirred overnight at 4 °C to complete the reaction. Next, MeHA was precipitated by acetone and washed three times with ethanol. Subsequently, precipitated MeHA was redissolved in Millipore water and was dialyzed for 5 days to remove the impurities. The purified MeHA was lyophilized for 3 days and stored in a desiccator for future use.^[^ ^31^, ^32^ ^]^

^1^H NMR and ^13^C NMR spectra were acquired at room temperature using an Ascend 300A spectrometer. Deuterium oxide (D₂O) was used as the solvent for the ^1^H NMR and ^13^C NMR analysis of DA, HA, DAHA, and MeHA. For methacrylic anhydride (MAA) analysis, chloroform‐D1 was used as the solvent. For the ^1^H NMR and ^13^C NMR measurements, sample solutions (1 mL) were transferred into 5 mm NMR tubes. DA, HA, DAHA, and MeHA were prepared at concentrations of 4 and 20 mg mL^−1^ in D₂O for ^1^H NMR and ^13^C NMR analyses, respectively. For the ^13^C NMR analysis of MAA, a mixture containing 5% (vol/vol) MAA in chloroform‐D1 was prepared. Subsequent data analysis was conducted using the TopSpin 4.3.0 software program.

### HMN Fabrication

To make the DAHA.MN array, first, lyophilized DAHA (50 mg) was dissolved in DI water (1 mL) and the solution pH was increased to 8 using 1N NaOH. The polymeric solution was then quickly transferred onto a 10 × 10 silicone MN mold (Micropoint Technologies Pte Ltd). To remove the bubbles trapped inside the silicone MN mold tip, centrifugation was done at 7700 rpm for 5 min. Next, the mold was left in the nitrogen desiccator at room temperature for 48 h for the composite hydrogel to be dried.

To fabricate the MeHA‐PR MN array, lyophilized MeHA (50 mg), MBA (1.5 mg), and Photoinitiator (PI, 1.5 mg), were dissolved in DI water (1 mL). After the solution was constantly stirred for 30 min, 10 uL of PR from a prepared stock concentration of 10 mm were added to the solution and the solution was stirred at room temperature for another 30 min. The MeHA‐PR solution was then poured onto a 10 × 10 silicone MN. To remove the bubbles trapped inside the silicone MN mold tip, 2 min vacuum was sufficient. Next, the mold was left in the nitrogen desiccator at room temperature for 72 h. Finally, the dried hydrogel was peeled off easily from the mold and was kept in the desiccator.

### Flexible Electrode Fabrication

To fabricate the flexible electrode in the cleanroom, the biocompatible and flexible polyimide (PI) sheets (purchased from DuPont Kapton) were first cleaned with acetone and isopropyl alcohol (IPA) and then attached to a silicon wafer (used as a holder to provide rigidity for the following steps). First, the AZ 4620 resist was spin‐coated on the wafer as an adhesive layer to attach the PI to the wafer, followed by PI attachment and baking. Then, e‐beam evaporation of Ti (20 nm), Au (175 nm), and Cr (20 nm) was performed to coat the PI with three‐layer metals, respectively. To pattern the electrode materials, the photolithography process was performed using the Ma‐N 1410 negative tone photoresist. The resist was spin‐coated and baked, followed by UV‐lithography and exposure using a prepared photomask and SUSS MA6 mask aligner and developing the UV resist and hard‐bake. The electrode metal layers were then patterned by a wet‐etching process using Chromium etchant, Gold Etchant TFA, and diluted 1:10 HF solution for Titanium etching. After the wet‐etching process, the photoresist was removed (used solvent process followed by oxygen plasma). To protect the designed unexposed area of the electrodes, the SU‐8 photoresist was used as a protective layer. For this process, SU‐8 was spin‐coated on the surface of patterned PI electrodes and pre‐baked, followed by another round of Photolithography using the SUSS mask aligner system with a predesigned photomask and postexposure baking. The SU8 was then developed using Propylene glycol methyl ether acetate (PGMEA). To have a pseudo‐reference electrode, the surface of the area designed for the reference electrode was coated with silver/silver chloride using electroplating of silver followed by chlorination of the coated film. The integration of the fabricated HMN and the fabricated flexible electrode was achieved through a straightforward process of pressing the electrode onto the HMN after the HMN application on the skin. The HMN‐Flex was then fixed on the skin using Tegaderm tape. The adhesive properties of DA facilitated a secure bond between the HMN and the electrode.^[^ ^66^, ^67^ ^]^

### Functionalizing the Flex Electrode with VAN/GEN Aptamer Probe

To modify the working electrode (WE) of the flexible electrode with aptamer, the previously reported protocols with slight modifications were used.^[^ ^18^, ^30^, ^68^ ^]^ Briefly, the electrodes were first cleaned by CV scanning in 0.5 m NaOH from −0.35 to −1.35 V with a scan rate of 0.1 V s^−1^, for ten cycles. Then the electrode is cleaned in 0.5 m H_2_SO_4_ by doing CV for ten cycles in the potential window of −0.35 to 1.5 V and the scan rate of 0.1 V s^−1^ for ten cycles. The electrode is then rinsed with DI water and incubated with the aptamer solution overnight (16 h). To make the aptamer solution, 1 µm of the aptamer is mixed with 100 µm of TCEP in the buffer (1X PBS + 2 mm MgCl_2_) and incubated (to get reduced) in the dark for 1 h. The WE electrodes are then covered with the aptamer solution overnight in the dark and in a humid chamber, followed by coating the surface with 1 mm MCH (made in the buffer) for 3 h. The modified electrodes were then rinsed and stored in the buffer at 4 °C in the dark until use. The sequence of the aptamers are provided in Table S3.

### In Vitro Validation of the Fabricated Flex Sensor and PR System

The in vitro testing of the fabricated Flex electrodes for detecting VAN/GEN was performed in buffer (1X PBS + 2 mm MgCl_2_) spiked with different concentrations of VAN/GEN. To determine the signal‐on and signal‐off frequencies for each of these aptamers, the aptamer‐modified Flex electrode was first dipped in a blank buffer and scanned (via SWV) at a range of frequencies (Ref). Guided by the literature, for the VAN aptamer, the frequencies of 1, 5, 10, 30, and 100 Hertz were tested while for the GEN aptamer 10, 30, 50, 100, and 300 Hertz were tested. The same electrodes were then dipped in buffers spiked with higher concentrations of VAN/GEN and were subjected to SWV at the stated frequencies. The sensor response at each target concentration and for each frequency was calculated via (I – I_0_) / I_0_, where “I” is the observed current (SWV peak height) at each condition, and “I_0_” is the observed current in the blank buffer. As a result, the frequency of 5 and 100 were selected as the signal‐off and signal‐on for the VAN aptamer. The frequencies of 10 and 300 Hertz were also selected as the signal‐off and signal‐on for the GEN aptamer. Afterward (and for the rest of the experiments), the sensor's response is defined as (I – I_0_) / I_0_, where the “I” represents the dual‐frequency current (I _signal‐on_ / I _signal‐off_) at the experimental condition and “I_0_” represents the dual frequency current (I _signal‐on_ / I _signal‐off_) of the (0 µm) buffer. To validate the selectivity of HMN‐Flex for VAN/GEN the aptamer‐functionalized Flex electrodes were subjected to non‐specific targets commonly present in ISF and their responses were recorded. These tested non‐specific targets are Ascorbic Acid (AA) (0.5 mm), Glucose (Glu) (10 mm), MgCl_2_ (0.5 mm), Uric acid (UA) (0.5 mm), GEN (10 and 50 µm for the VAN Flex sensor), and VAN (10 and 20 µm for the GEN Flex sensor), Doxorubicin (DOX) (100 ng mL^−1^), and Tobramycin (TOBI) (10 µm).

### Testing the HMN‐Flex and HMN‐pH Ex Vivo

For the ex vivo experiments of HMN‐Flex, porcine skins (pig ears skin) were trimmed to 1 cm × 1 cm squares, rinsed with DI water, and equilibrated with different concentrations of the biomarkers of interest (VAN/GEN) for 16 h. The HMN‐Flex patches were then applied on the skins equilibrated with different concentrations of the targets. To apply the HMN‐Flex, first, the HMN was inserted into the skin, followed by laying the Flex electrode on top of the HMN. After 5 min, the electrodes were scanned via SWV at the dual frequencies, and their currents were recorded based on which the sensor's response was calculated.

The performance of the HMN‐Flex under mechanical tension was also assessed by first applying the HMN‐Flex on a piece of target‐equilibrated porcine skin, then subjecting the porcine skin to cycles of bending and twisting and recording its response after each cycle. Additionally, we subjected the skin piece and its accompanying HMN‐Flex to 100 bends and twists, then recorded the response when the skin piece was in the normal condition, at the bent condition, or at the twisted condition. The response after these mechanical changes was compared to before applying the mechanical tensions and is reported as the Change %.

For the ex vivo testing of HMN‐pH, 2 cm × 2 cm of porcine skin was equilibrated with standard buffers from 7 to 8 pH for 24 h. The HMN‐pH patches were then inserted into the porcine skin and underwent swelling for 15 min. The HMN‐pH patches were then removed from the skin and pictures of MN arrays were taken. Finally, based on the RGB values acquired from the HMN‐pH pictures a calibration curve was plotted.

### Scanning Electron Microscopy (SEM) Imaging

To visualize the DAHA‐HMN and MeHA‐HMN arrays, the patches were prepared, coated with a 2 nm thick layer of gold, and imaged using a Hitachi SU5000 FESEM. To visualize and analyze HMN‐Flex and HMN‐pH pore sizes, thin films were prepared and allowed to swell in water for 5 min. The films were then snap‐frozen with liquid nitrogen and freeze‐dried for 48 h followed by being coated with a 2 nm thick layer of gold and imaged using a Hitachi SU5000 FESEM.

### Swelling Ratio Analysis

The prepared DAHA‐HMN and MeHA‐HMN patches were prepared and weighed (W_0_). A 1.4 wt.% agarose was prepared. The patches were inserted into the agarose for 2 min. The patches were then removed and immediately weighed (W_T_). To calculate the swelling ratio, the following formula was used:

(1) $$\mathrm{Swelling}\;\mathrm{ratio}\;\left(\%\right)=\frac{w_{T}-w_{o}}{w_{o}}\times 100\%$$

### Mechanical Strength

The mechanical strength of DAHA‐HMN and MeHA‐HMN patches was measured using an Instron 5548 micro tester equipped with a 500N compression loading cell. For each test, the HMN patch was placed flat on its backside (tips facing upward) on a compression platen. The distance between two platens was set to 1.5 mm. A vertical force was applied (at a constant speed of 0.5 mm min^−1^) by the other platen. The compression loading cell capacity was set to 70 N. The load (force; N) and displacement (distance; mm) were recorded by the testing machine every 0.1 s to create the load‐displacement curve.

### PR Release Testing

To investigate the possible release of PR from HMN‐pH, three HMN‐pH patches were fabricated (using the aforementioned method). These patches were then placed into a 1.4% Agarose (which mimics skin) for 15 min and subsequently removed. The Agarose gel was then heated for 1 h at 100 °C to liquefy. Another 1.4% Agarose gel, containing 100 µm PR (the same concentration as the patches), was prepared as a positive control and also liquefied using heat. The absorbance spectra of PR within the wavelength range of 375–505 nm for these two samples were measured (Figure S). Since the PR's peak absorbance had happened at 440 nm, the percentage release for PR was quantified using the following: Absorbance at 440 nm for HMN‐PR / Absorbance at 440 nm for the positive control * 100.

### Biocompatibility Validation by Looking at Cell Viability

The biocompatibility of the three HMNs was assessed using a Methylthiazolyldiphenyl‐tetrazolium bromide (MTT) assay with mouse fibroblast cells (NIH/3T3). 50000 cells/well at a total volume of 200 µL were seeded in a 24‐well plate. The biocompatibility of the DAHA and MeHA‐PR hydrogels was assessed by adding the hydrogel solutions (10 µL) into the wells, with a 24‐h incubation. DMEM medium (10 µL) was used as a control. Next, a 5 mg mL^−1^ MTT stock solution (20 µL) was added to the wells and incubated at the dark for 3 h. DMSO was used to break up the cells (300 µL) and samples were transferred to a 96‐well plate for UV absorbance measurements at 540 nm. The percentage viability is reported as UV absorbance in hydrogel well / UV absorbance at control well * 100.

### Histology Imaging

To visualize the needle cavities on the rats, the common method of H&E staining was used. HMN‐Flex and HMN‐pH patches were applied to the shaved dorsal rat skin for 5 min. The rat was euthanized after removing the patches and the skin sections were cut and washed with 0.9% NaCl solution. The samples were fixed with neutral buffered 10% formalin for 24 h, then stored in 70% ethanol and refrigerated. Fixed skin samples were cryopreserved in 15% sucrose/PBS solution at 4  C overnight. Following cryopreservation, samples were briefly rinsed in PBS to remove residual sucrose. Samples were mounted on cork using OCT (TissueTek), frozen in isopentane cooled by liquid nitrogen, and stored at −80  C. 10‐micron sections were cut using a cryostat maintained at −20  C and mounted on microscope slides. Hematoxylin and eosin (H&E) stains were used to identify the basic morphology of skin samples. Slides were stained with Harris‐modified hematoxylin (Sigma, HHS32) for 30‐s, washed in distilled water, and counterstained with 1% Eosin Y (Sigma, E4009) for 2 min. Slides were washed in distilled water, dehydrated in 75% and 95% ethanol, and cleared in Xylene prior to mounting with Permount mounting medium (Fisher Scientific, SF15). Images were acquired using a Cytation‐5 multimode imager (Agilent). Images were obtained at 20X magnification and stitched together using the Gen5 software (Agilent).

### In Vivo (Animal) Experiments

In vivo experiments were done following the Guidelines for the Care and Use of Laboratory Animals and the Animal Welfare Act Regulations; all protocols were approved by the University of Waterloo Institutional Animal Care and Use Committee. To examine the performance of HMN‐Flex and HMN‐pH, Male Sprague Dawley rats (Charles River, 200–500 gr) were intravenously injected with different dosages of VAN (15 and 45 mg kg^−1^ made in saline) or GEN (50 and 100 mg kg^−1^ made in saline). Before applying the HMN‐Flex patches, the rats were subjected to isoflurane anesthesia, their skin was shaved, treated with hair removals, wiped, and cleaned with ethanol. Similar to ex vivo experiments, the HMN patch was first inserted on the rat's dorsal skin followed by laying the Flex‐electrode over the HMN and fixing them with Tegaderm tape. At this point, the rat's venous blood was collected to draw the serum VAN/GEN levels before antibiotic injection. 5‐min past the HMN‐Flex application, we started to interrogate the HMN‐Flex through SWV and recorded the resulting response. We continued the interrogation until a stable response was observed (typically happened 15 min after HMN‐Flex application) and considered the response as the baseline response (before antibiotic injection). Then the rats were intravenously injected with the antibiotic regimens of interest, and the HMN‐Flex was interrogated every 5–10 min up to 120 min (for VAN detection) and 90 min (for GEN detection) to record the response. In the case of GEN, since longer‐term measurements were required, the Flex electrode was removed, stored in the buffer, and reapplied 6 and 24 h postinjection (the performance of the stored electrodes was checked before their reapplication). The continuous measurements of VAN and GEN were done up to 120 and 90 min because our animal protocol did not allow keeping the animals under anesthetic conditions for longer than 120 min. Throughout the process, the rat's venous blood was collected every 30 min, and their VAN/GEN levels were measured based on which the serum AUC and C_max_ were calculated. The VAN/GEN level at T = 0 (right after injection) was extracted based on modeling the behavior of other concentrations over time. To validate the HMN‐Flex for in vivo VAN and GEN detection, a total of two rats (each injected with two different VAN/GEN regimens) were used.

For in vivo multiplexing of the GEN and pH, the same steps and single‐plex detection were followed, where both HMN‐Flex and HMN‐pH were applied on the rat's dorsal skin and their responses were simultaneously recorded.

### Pharmacokinetic (PK) Analysis

Two distinct PK models were utilized for this purpose: one for describing antibiotic concentrations in serum, using a 1‐compartment PK model with linear elimination, and another for ISF, consisting of three compartments in series. The model fitting process was carried out using the first‐order conditional estimation method with interaction (FOCEI) routine in Nonmem v7.4, and subsequent PK profiles were simulated from the fitted models using the mrgsolve package in R. AUC and C_max_ values were directly derived from the simulated PK profiles, and the correlation between these PK parameters in serum and ISF was assessed using the Spearman correlation test.

### VAN, GEN, and pH Serum Analysis

To isolate serum from the collected blood samples, they were first clotted by being left undisturbed at room temperature for 30 min, followed by centrifugation at 2000 g and at 4 °C for 10 min. The resultant blood serum was collected and stored at −20  C until further use.

To analyze the concentration of VAN in the collected serum samples we used the commercially available VAN ELISA kit from Abcam (ab 285230) following the provided instruction. GEN levels in serum were measured via the liquid chromatography‐mass spectrometry (LCMS) technique. For this purpose, the Agilent 1260 Infinity and 6130 single quadrupole LCMS instrument, was equipped with Agilent ZORBAX Eclipse AAA (4.6 × 75 mm, 3.5 micron) column. The mobile phase was running in a gradient manner with initial conditions starting with 70:30:0.1% H_2_O: (acetonitrile) ACN:(Formic acid) FA v/v/v pumped through the system at 0.2 mL min^−1^ for 3.5 min. Then the percentage of ACN was linearly increased to 95% over 1.5 min pumped at 0.5 mL min^−1^. The detection was set to UV 254 nm and single ion monitoring (SIM) m/z 478 (corresponding to [gentamicin M+H]^+^). The standard curve was established using the matrix‐matched method. Briefly, 10 000 µm stock solution of GEN standard was prepared by dissolving GEN in ultra‐pure water (UPW) which is followed by serial dilution to 1250 and 500 µ. Then, 10 µL of the standard solution was added to 10 µL of blank serum and further diluted 10 times with 80 µL UPW to give 125 and 50 µm matrix‐adjusted standard solution. The resulting matrix‐adjusted standard solutions were then filtered through a 10 kDa molecular weight cut‐off centrifuge filter spined at 10 000 g for 15 min. The standard curve was generated by injecting different volumes (e.g., 10.0, 7.5, 5.0, 2.5, 1.0 µL) from 125 and 50 µm matrix‐adjusted standard solution. For the serum samples, similarly, 10 µL serum was diluted ten times with 90 µL UPW and then filtered with the same procedure.

To analyze pH of serum, 9 µL of serum from each timepoint was mixed with 1 uL of 1 mm PR. Before and after the addition of PR, the sample's absorbance was measured using UV–vis Spectroscopy. For each time point, the difference spectrum of before and after PR addition was calculated and a difference curve was plotted. Using pH buffers of 7, 7.5, and 8, the standard pH curves were derived and used to interpolate the serum‐PR difference curve.

### Statistical Analysis

All experiments were performed with at least three replicates with the error bars showing the standard deviation or standard error of the mean. For each experiment, the number of replicates and error bars have been reported in their corresponding figure captions. The statistical analysis was performed using GraphPad Prism software. In Figure 2b,c to assess whether the difference between non‐specific targets and the specific targets for VAN and GEN are significant, we have performed a one‐way analysis of variance (ANOVA) and post hoc Tukey tests. The result for one‐way ANOVA (*p* = 0.05) was found significant (*p* < 0.0001), and the post hoc Tukey test also presented significant differences in the response to the specific targets compared to the nonspecific ones where the significances are shown in GP style (0.1234 (ns), 0.0332 (^*^), 0.0021 (^**^), 0.0002 (^***^), *p* < 0.0001 (^****^)). To compare the swelling of DAHA and MeHA HMN patches, in Figure 3c, three replicates were performed per condition and data is shown as the mean ± SD, *n* = 3. *p*‐values were determined using one‐way ANOVA, *p* < 0.05 (^*^), and *p* < 0.01 (^**^), and *p* < 0.001 (^***^). To validate the in vivo performance of HMN‐pH meter, in Figure 5, the sensor response was compared with the serum pH measurement with three replicates per each sensor or blood measurement, and data is shown as the mean ± SD for all measurements. *p*‐values were determined using one‐way ANOVA, *p* > 0.05 (n.s.). To report the correlation between ISF and serum GEN / VAN C_max_ and AUCes (Figure 5c,d), the Pearson correlation coefficient has been used and the correlation coefficient “r” is reported where the significances are shown in GP style (0.1234 (ns), 0.0332 (^*^), 0.0021 (^**^), 0.0002 (^***^), *p* < 0.0001 (^****^)). To examine the mechanical stability (Figure S6, Supporting Information), significance was determined through one‐way ANOVA with Tukey post hoc test (where the, *p* = 0.21 for the VAN sensor and *p* = 0.81 for the GEN sensor, and ns mean non‐significant (*p* < 0.05 is considered significant).

The limit of detection (LOD) for VAN and GEN sensors was calculated using the below formula:

(2) $$\mathrm{LOD}=\frac{3\times \mathrm{Standard}\;\mathrm{deviation}\;\mathrm{of}\;\mathrm{the}\;\mathrm{fitted}\;\mathrm{line}}{\mathrm{Slope}\;\mathrm{of}\;\mathrm{the}\;\mathrm{fitted}\;\mathrm{line}}$$

## Conflict of Interest

The authors declare no conflict of interest.

## Author Contributions

F.K., P.G., and M.A.S. contributed equally to this work. F.K., P.G., M.A.S., and M.P. discussed the idea. F.K. did the VAN and GEN sensor development and validation, as well as their ex vivo and in vivo testing. P.G. performed pH assay development and validation and HMN fabrications/characterizations. M.A.S. contributed to the initial aptamer assay development. Y.Z., A.S., S.S., H.Z., F.R. helped with validation of animal experiments. S.S performed the NMR experiments. P.C. performed the PK modeling. M.S. fabricated the flexible electrodes. F.K. led the manuscript writing while P.G., M.A.S., Y.Z., P.C., M.S., A.E., and M.P. contributed to editing.

## Supporting information

Supporting Information

## Data Availability

The data that support the findings of this study are available from the corresponding author upon reasonable request.
