# Supplementary material for: Integrated Electrochemical Aptamer Biosensing and Colorimetric pH Monitoring via Hydrogel Microneedle Assays for Assessing Antibiotic Treatment
Source: Adv Sci (Weinh). 2024 Sep 9;11(41):2309027. doi: 10.1002/advs.202309027 (PMC11538706; doi:10.1002/advs.202309027)
Supplement: Supplementary file 1 — Supporting Information [file ADVS-11-2309027-s001.docx]

**Integrated Electrochemical Aptamer Biosensing and Colorimetric pH Monitoring via Hydrogel Microneedle Assays for Assessing Antibiotic Treatment**

*Fatemeh Keyvani^1^, Peyman Ghavaminejad^1^, Mahmoud Ayman Saleh^2^, Mohammad Soltani^1^, Yusheng Zhao^3^, Sadegh Sadeghzadeh^1^, Arash Shakeri^3^, Pierre Chelle^3^, Hanjia Zheng^1^, Fasih A. Rahman^4^, Sarah Mahshid^2^, Joe Quadrilatero^4^, Praveen P. N. Rao^3^, Andrea Edginton^3^, Mahla Poudineh^1^ ^*^*

Department of Electrical and Computer Engineering, Faculty of Engineering, University of Waterloo, Waterloo, ON N2L 3G1, Canada

Department of Bioengineering, McGill University, 815 Sherbrooke St W, Montreal, Quebec H3A 0C3, Canada

School of Pharmacy, University of Waterloo, Waterloo, ON N2L 3G1, Canada

Department of Kinesiology and Health Sciences, University of Waterloo, Waterloo, ON N2L 3G1, Canada.

*Correspondence to mahla.poudineh@uwaterloo.ca

**This PDF includes:**

**Figure S1:** ^1^H NMR spectra of DAHA

**Figure S2:** ^13^C NMR spectra of DA, DAHA, and HA

**Figure S3:** The schematic for fabricating the flexible electrode.

**Figure S4:** ^1^H NMR spectra of MeHA

**Figure S5:** ^13^C NMR spectra of MAA, MeHA, and HA

**Figure S6:** The impact of using the “dual frequency” method

**Figure S7**: PR release investigation

**Figure S8**: HMN-Flex response under mechanical tension

**Figure S9**: Hydrogel biocompatibility

**Figure S10:** VAN response for four rats

**Figure S11:** VAN concentration profile in ISF and serum

**Figure S12:** VAN ELISA kit calibration curve used for measuring serum VAN

**Figure S13**: PK modeling

**Figure S14:** Calibration curve used to interpolate HMN-Flex response to GEN

**Figure S15**: LCMS data for preparing the GEN calibration curve in serum

**Figure S16**: A representative calibration curve for GEN

**Figure S17**: Representative LCMS data for a rat at different time points

**Figure S18:** GEN concentration profile in ISF and serum for 6 rats

**Figure S19**: HMN-Flex response after injecting a rat with blank saline

**Table S1**: Recent studies on electrochemical aptamer-based sensors for TDM

**Table S2:** The ISF C_max_ and AUC associated with different dosages of VAN/GEN

**Table S3:** The sequence of the GEN and VAN aptamers

Supplementary Figures


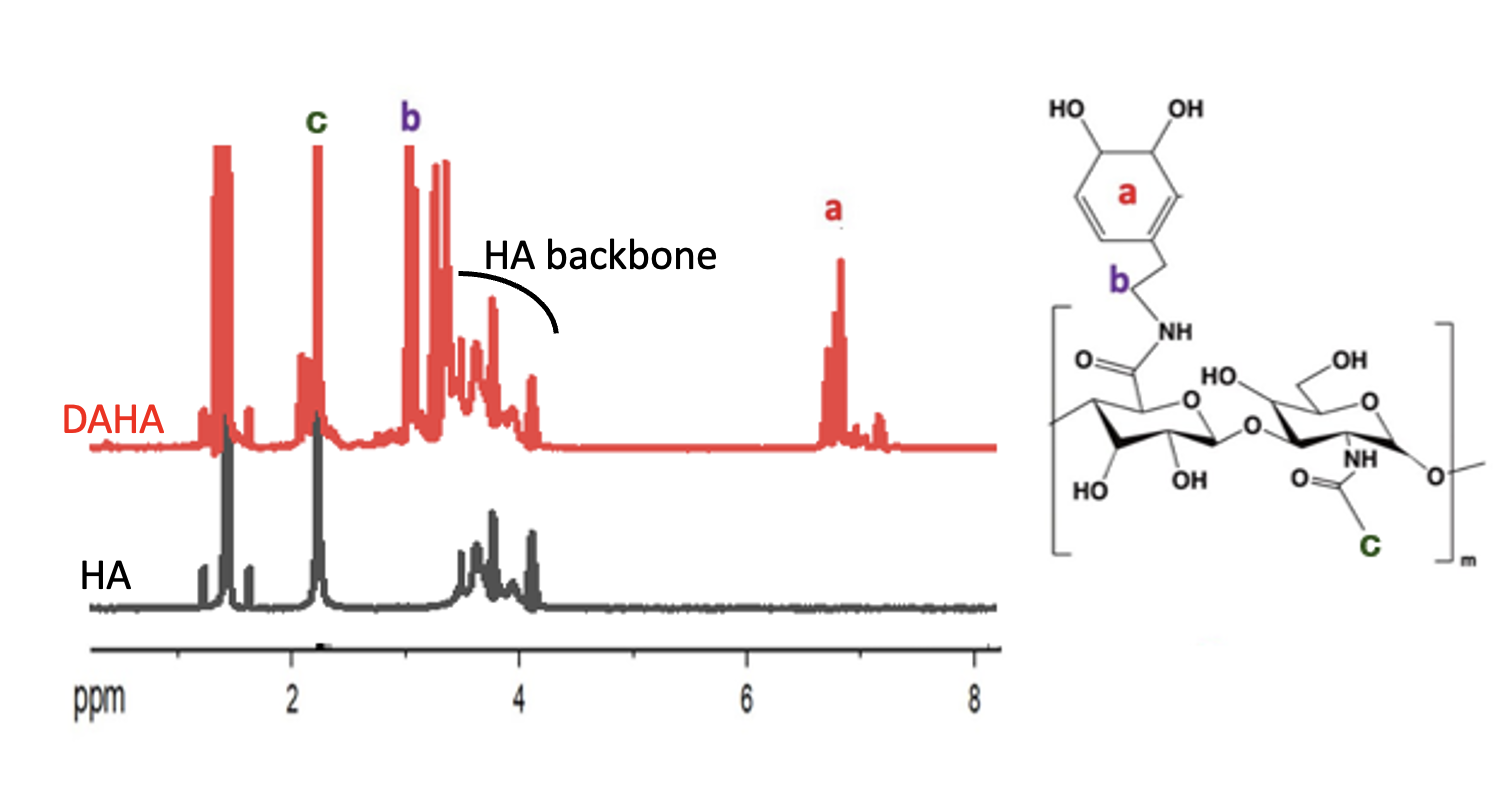


**Figure S1.** Comparison of ^1^H NMR spectra of HA (black) and DAHA (red), region (a) or the peaks appearing between 6.5 and 7.0 ppm attributed to the protons at benzene ring, peak at around 3 ppm attributed to the proton at (b) site or methylene group of dopamine, and the peak (c) corresponds to the proton of the N-acetyl group on HA. The peaks from 3-4 ppm represent the protons on the HA backbone.


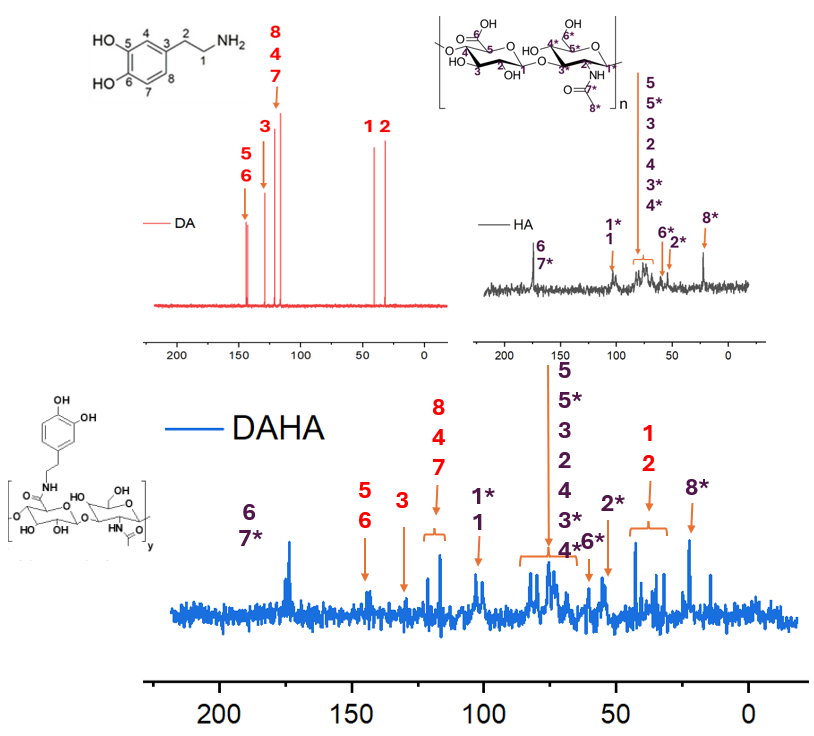


**Figure S2.** ^13^C NMR spectra of DA, HA, and DAHA in D_2_O and the corresponding peaks.


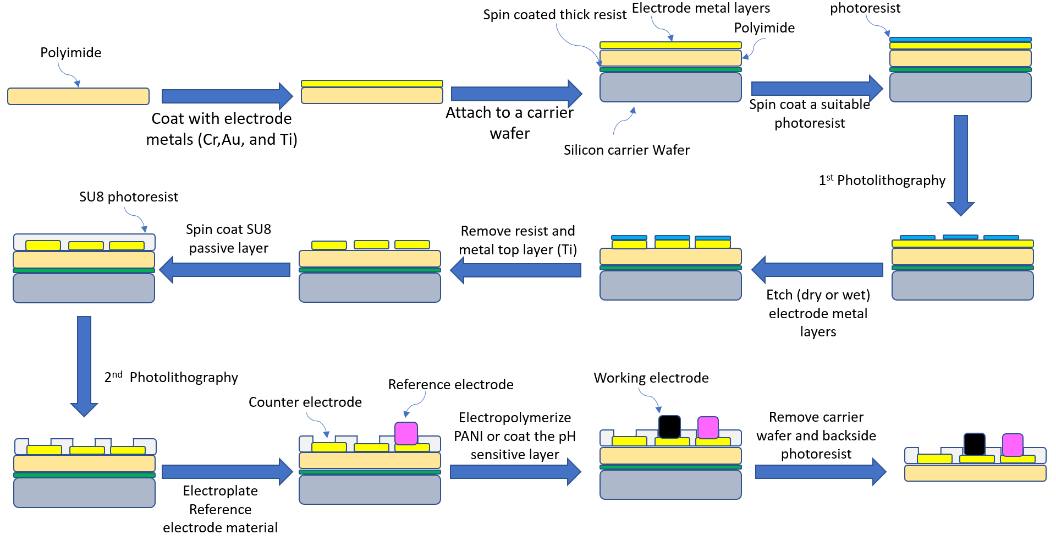


**Figure S3.** The schematic presents the process followed for fabricating the flexible electrode.


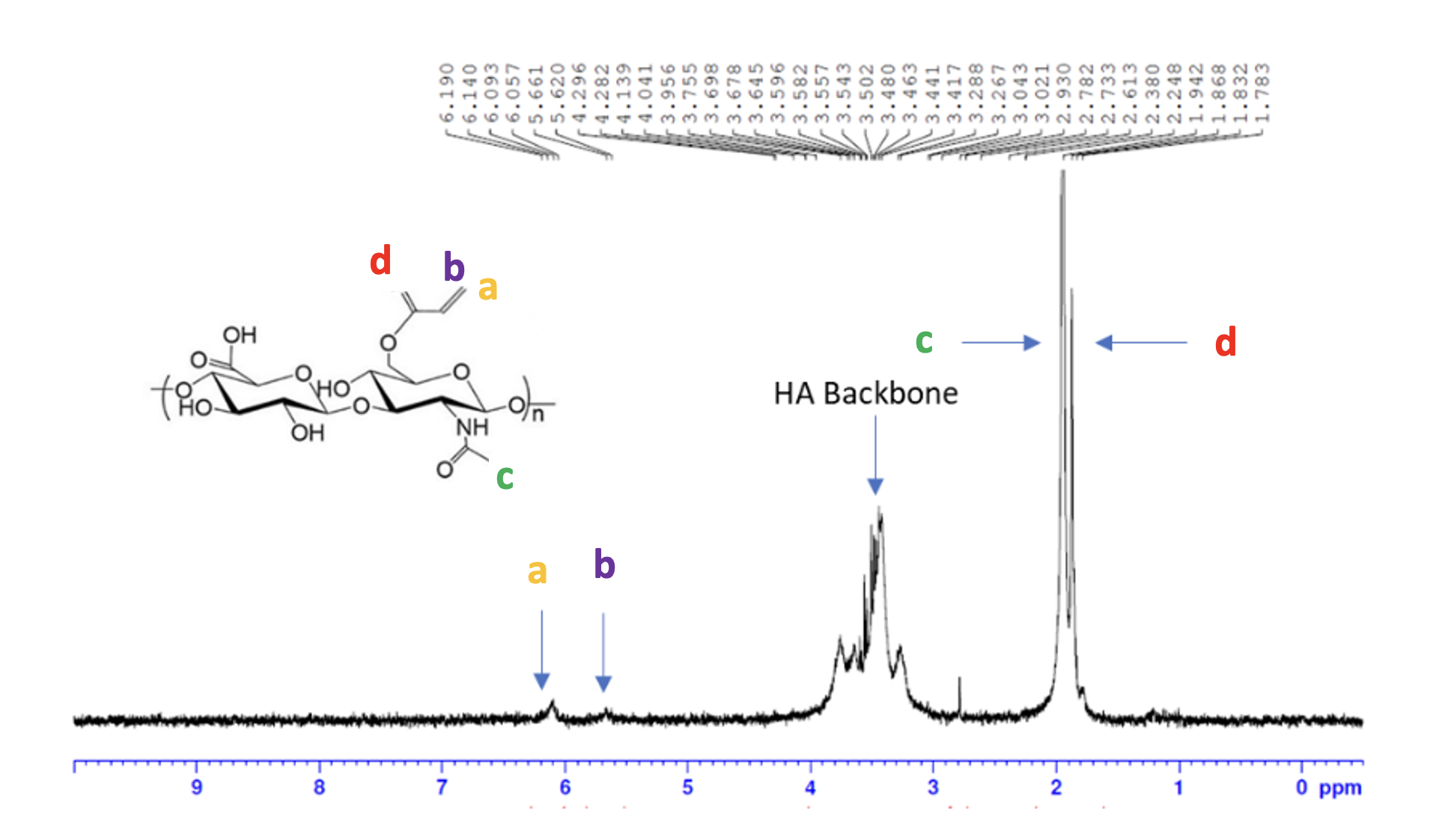


**Figure S4.** ^1^H NMR spectra of MeHA. The proton peaks at 5.7 and 6.1 ppm ((a) and (b) regions) correspond to the protons on the methacrylate's alkene. The peaks from 3 to 4.2 ppm are the signals from the HA backbone (refer to figure S1). The peak at 1.9 ppm represents the proton peak of the methyl group on the methacrylate (region (d)), while the peak at 2 ppm corresponds to the protons on the N-acetyl group in the HA backbone (region (c)).


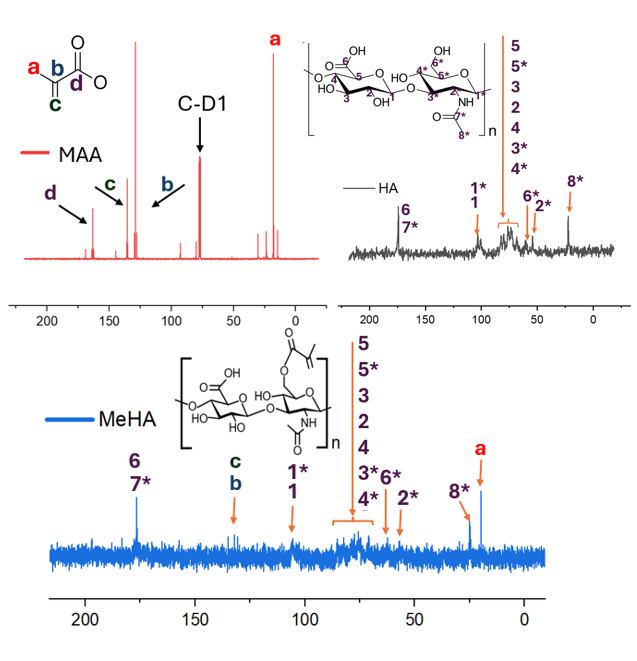


**Figure S5.** ^13^C NMR spectrum of MAA, HA, and MeHA in D_2_O and the corresponding peaks.

**Figure S6.** The impact of using the “dual frequency” method on improving the sensor’s response to VAN (a), and GEN (b). The connecting line shows the fit line. The sensitivity (the slope of the linear range of calibration curve) of VAN sensor when using the dual-frequency response and signal-on response is 2.3 and 0.63, respectively, demonstrating an improved sensitivity of 3.6 times higher. The sensitivity (the slope of the linear range of calibration curve) of GEN sensor when using the dual-frequency response and signal-on response is 2.64 and 0.16, respectively, demonstrating an improved sensitivity of 16.5 times higher.

a

c

b

**
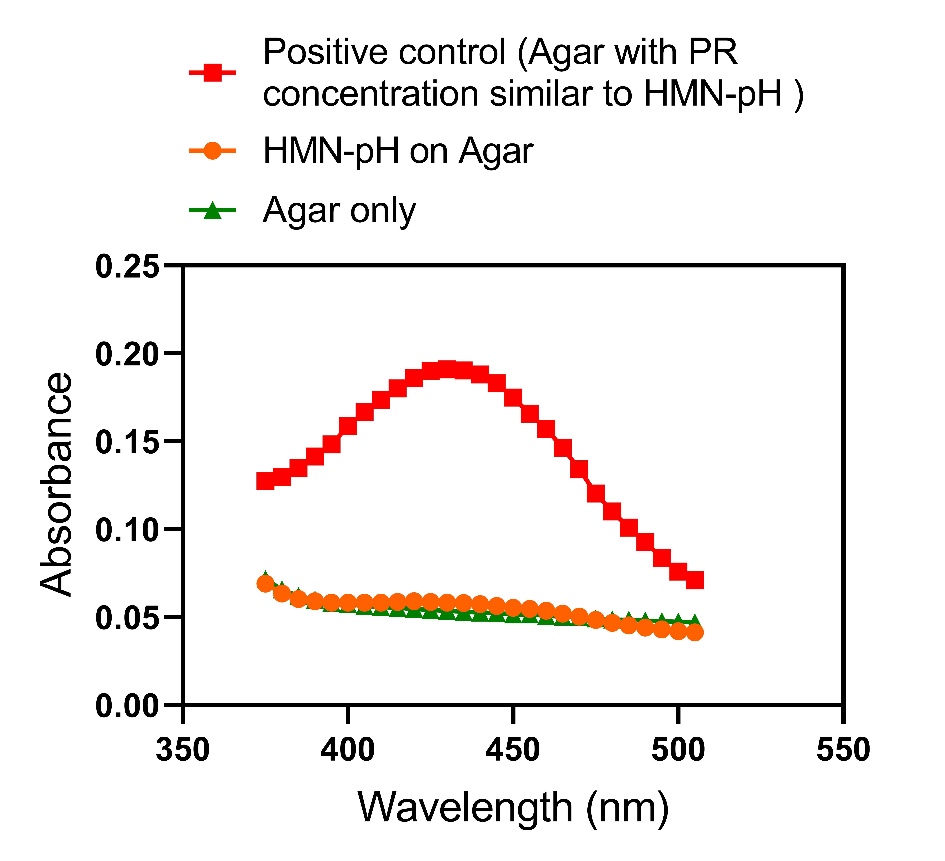
Figure S7.** To investigate the PR release from HMN-pH, the absorbance spectra of agar after applying the HMN-PR and removing it were obtained. The increase in absorbance at 440 nm (the peak absorbance for PR) of Agar with PR, HMN-pH, and agarose only are 0.188, 0.057, and 0.052, respectively, indicating a negligible (3%) PR release had happened.


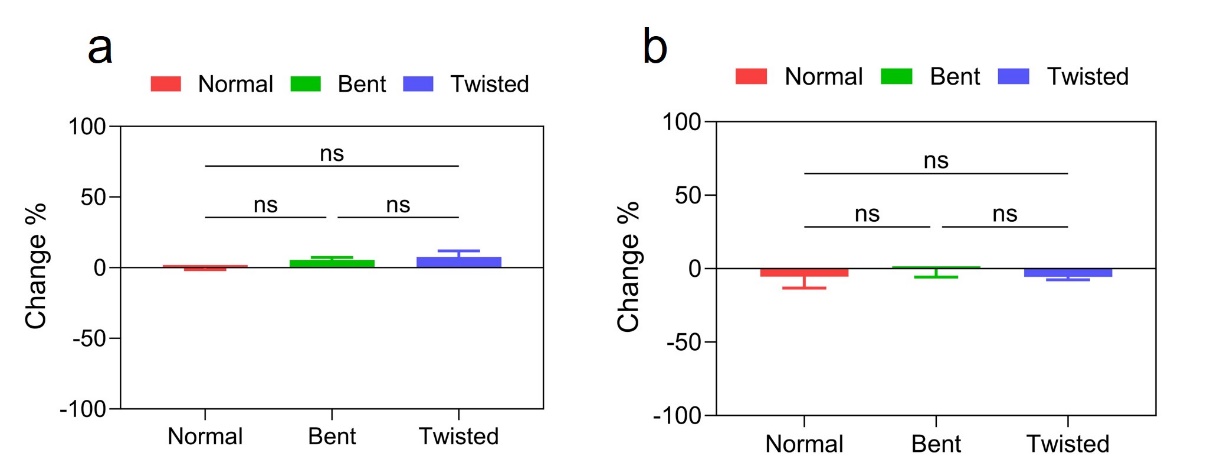


**Figure S8**. The HMN-Flex response under normal, twisted, and bent states in porcine skin equilibrated with 30 µM VAN (**a**) or 20 µM GEN (**b**) after 100 consecutive cycles of twisting and bending. Error bars represent the standard deviation of n = 3. Significance is determined through one-way ANOVA with Tukey post hoc test (where the P = 0.21 for VAN sensor and P = 0.81, and ns mean non-significant (p < 0.05 is considered significant).

**Figure S9.** The biocompatibility test for the DAHA and MeHA-PR hydrogels. The control well contained growth media only while the DAHA and MeHA-PR wells contained growth media with the respective hydrogels.

**Figure S10.** Graphs illustrating the HMN-Flex response to the fluctuating levels of VAN before and after intravenous injection of 15 mg/kg (a) and 45 mg/kg (b) VAN in addition to serum VAN concentrations (measured with conventional ELISA method, where ND is reported as 0) in rat#1. Graphs showing the interpolated VAN concentration from HMN-Flex response before and after intravenous injection of 15 mg/kg (c) and 45 mg/kg (d) VAN in addition to serum VAN concentrations in rat#2.

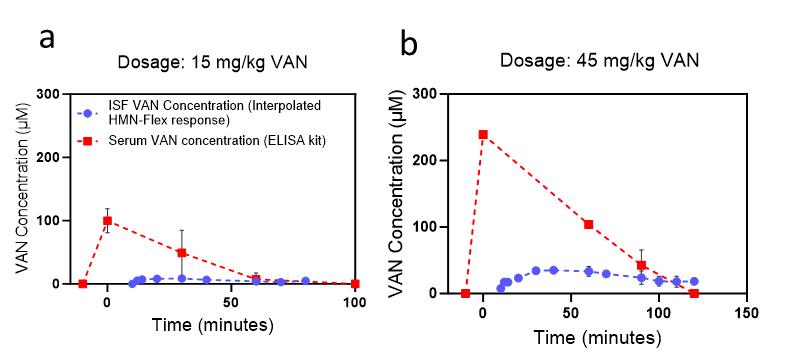


**Figure S11.** Figures showing the ISF VAN concentration (interpolated HMN-Flex response) to VAN when Rat#2 received the two different dosages of 15 (a) and 45 mg/kg (b) of VAN. n ≥3 and error bars show the standard deviation


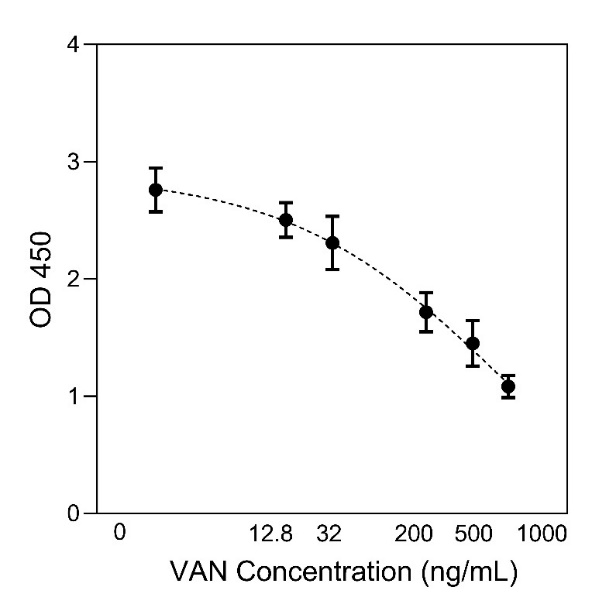
**Figure S12.** The calibration curve from the VAN ELISA kit. As per the product’s instruction, n=3 was used to draw the calibration curve, and the error bar shows the standard error of the mean. The X-axis shows the OD which is the optical density at 450 nm wavelength.


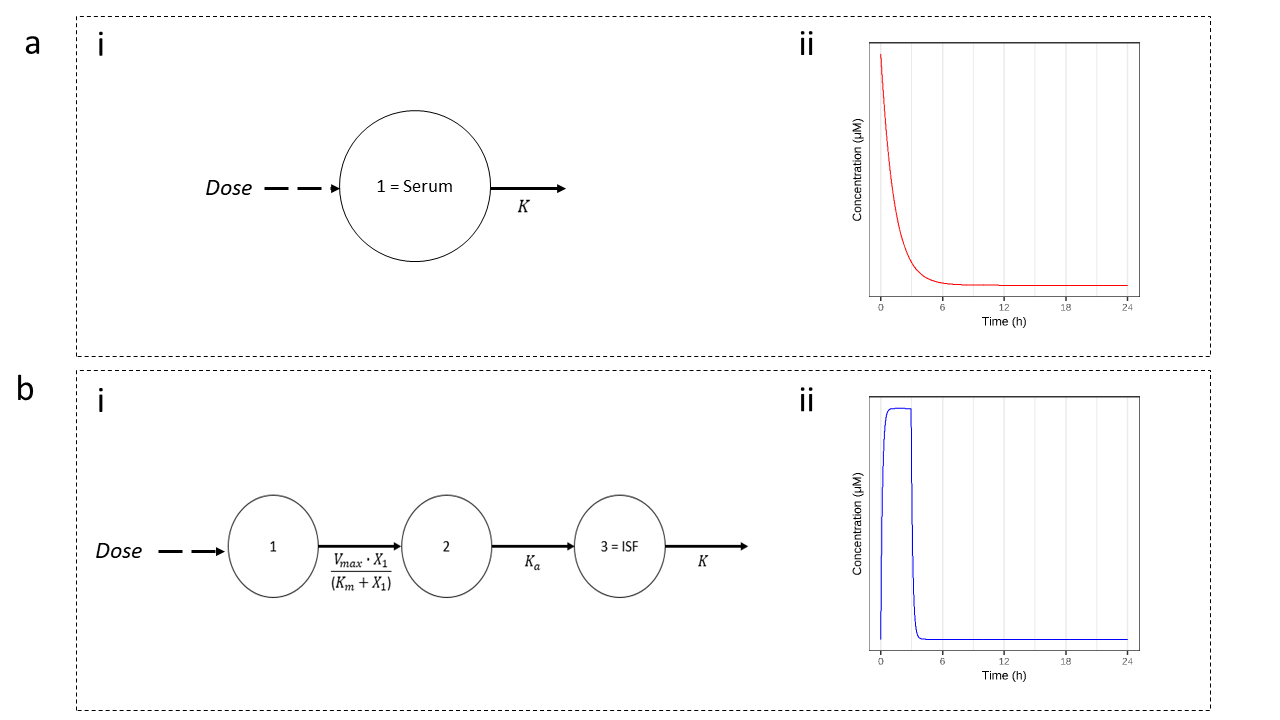


**Figure S13.** Representation of the PK model used to describe the concentration and drive C_max_ and AUC in serum (a, i). Illustration of a PK profile simulated from the PK model describing serum concentration (a, ii). Representation of the PK model used to describe the concentration and drive C_max_ and AUC in ISF (b, i). Illustration of a PK profile simulated from the PK model describing ISF concentrations (b, ii) .

**Figure S14.** Calibration curve used to interpolate HMN-Flex response to GEN.

**
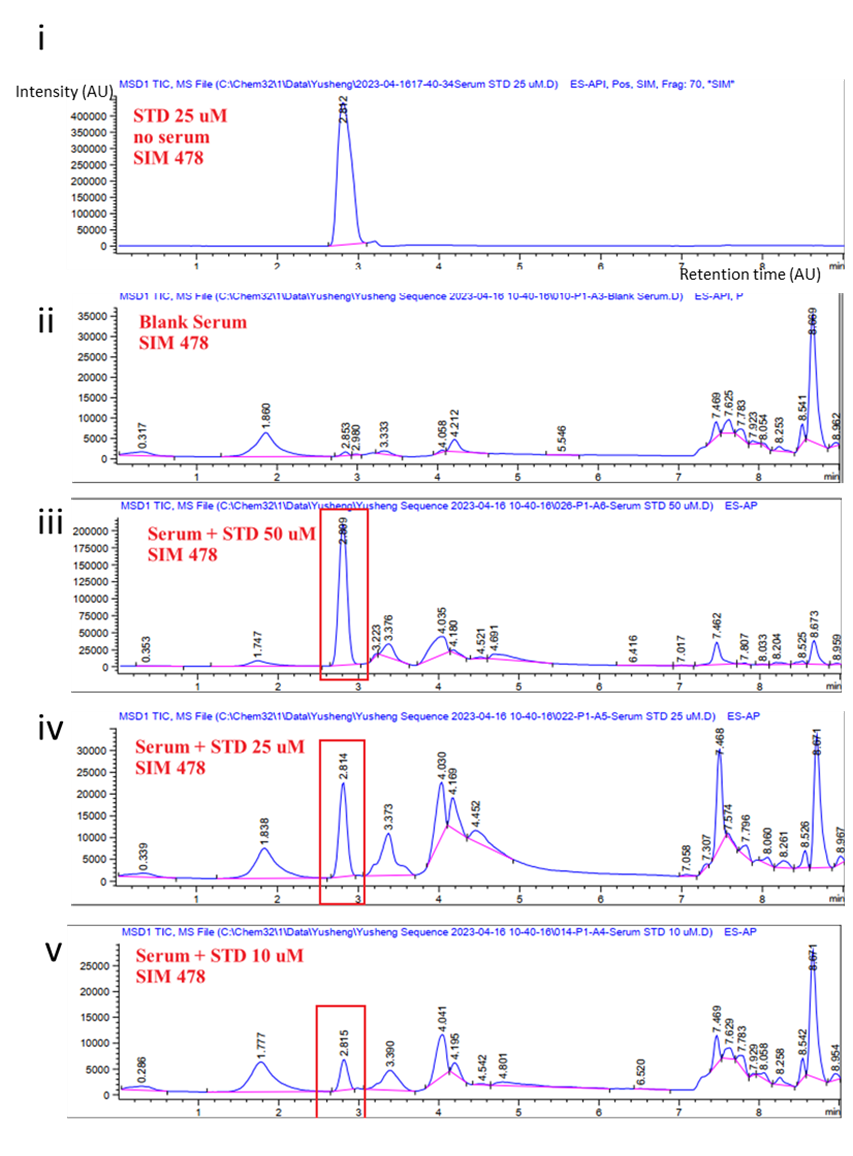
**

**Figure S15**. Representation of the SIM scans for a GEN sample (i), as well as a blank serum (ii), and serum spiked with 50 µM (iii), 25 µM (iv), and 10 µM (v) GEN with the peak outlined in red at 2.8 min retention time showing the GEN peak.

**Figure S16.** Graph showing a typical calibration curve drawn from spiked serum samples where the n=3, the error bars represent the standard deviation (the AUC es from left to right are: 927335.3 ± 53123.35, 1810987 ± 45059.36, 5773587 ± 85437.5, 6819443 ± 26221.3, 7503625 ± 172105), and the dashed line is the linear fit.


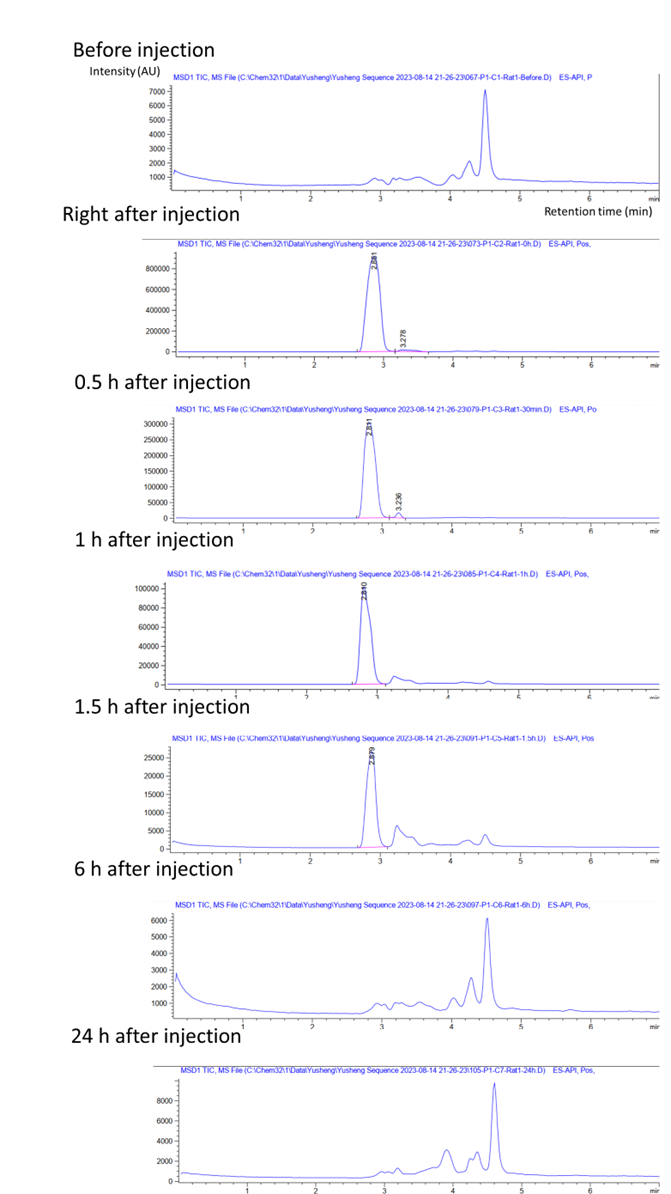


**Figure S17.** Representation of the SIM scans for serum samples collected from a rat (who received 50 mg/kg of GEN) before injection and at different time points after injection.


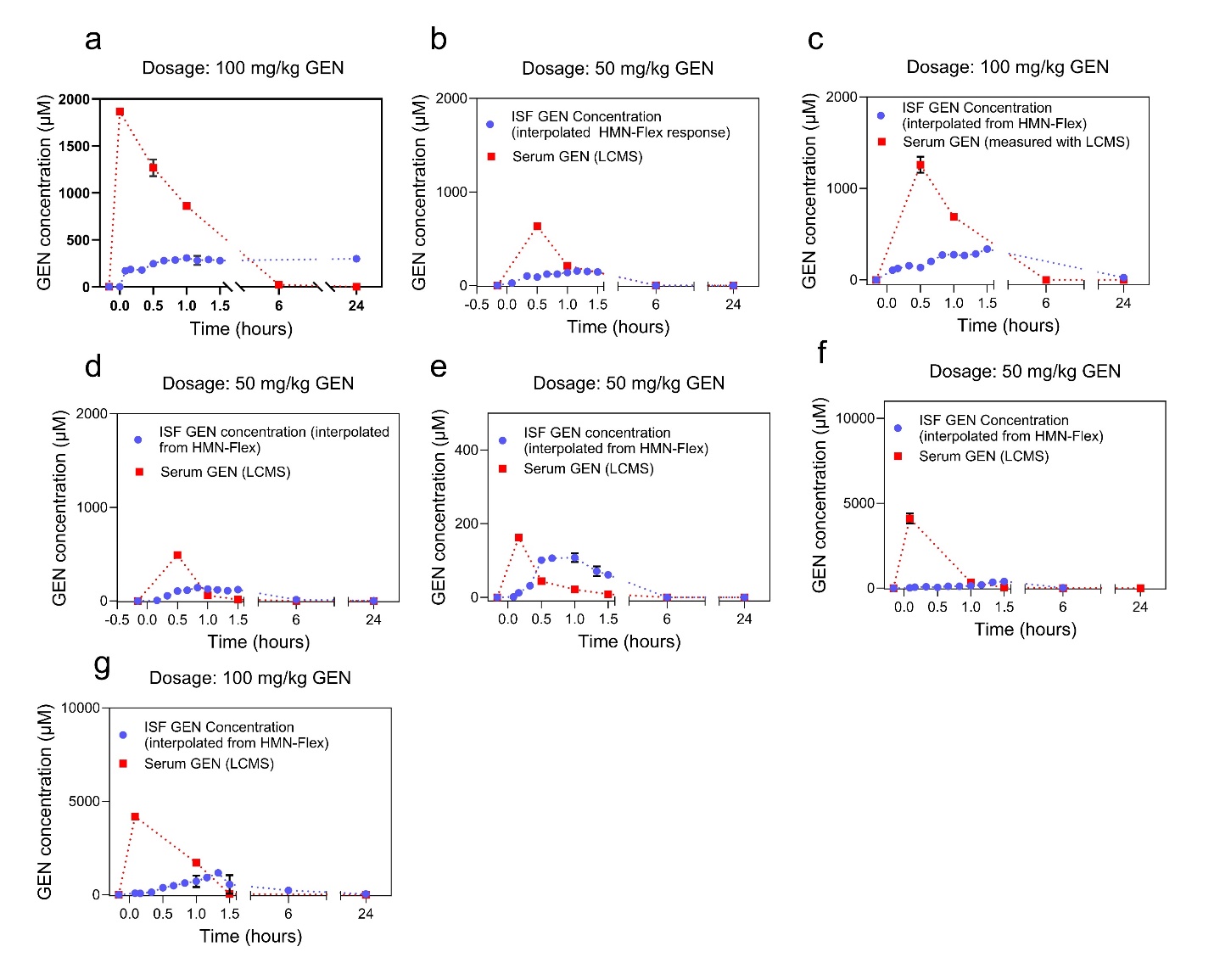


**Figure S18.** Graphs illustrating the interpolated GEN concentration from HMN-Flex response to the fluctuating levels of GEN before and after intravenous injection of 50 mg/kg and 100 mg/kg GEN in addition to serum GEN concentrations (measured with conventional LCMS method, where ND is reported as 0) in rat#1 (a), rat#2 (b, c), rat#3 (d), and rat#4 (e), rat#5 (f), rat#6 (g). n ≥ 3, and error bar shows the standard deviation.

**Figure S19.** The figure represents the HMN-Flex response after injecting a rat with blank Saline (void of VAN/GEN).

**Table S1.** A summary of recent studies on electrochemical aptamer-based sensors for TDM

| **References** | **Invasive application** | **In-vivo Durability** | **Directly contact between aptamer and skin/vein** | **Target drug** | **Body fluid** |
| --- | --- | --- | --- | --- | --- |
| [31] | Yes  (Inserting a catheter into the jugular vein) | 5 h | Yes | Vancomycin | Blood |
| [32] | Yes  (Inserting a catheter into the jugular vein) | 4 h | Yes | Tobramycin, Doxorubicin, Gentamicin, Kanamycin | Blood |
| [33] | Yes  (Inserting a catheter into the jugular vein) | 2 h | Yes | Irinotecan | Blood |
| [9] | Minimal (Solid Microneedle) | 1 h | Yes | Tobramycin, Irinotecan, Doxorubicin | ISF |
| [34] | Minimal (Solid Microneedle) | 1 h | Yes | Tobramycin | ISF |
| This work | Minimal (Hydrogel Microneedle) | 2 h for continuous measurements and reusable electrodes for up to 24 h. | No | Vancomycin, Gentamicin, In addition to pH. | ISF |

**Table S2.** The C_max_ and AUC es calculated from the HMN-Flex response in different rats injected with the different dosage of VAN or GEN.

**
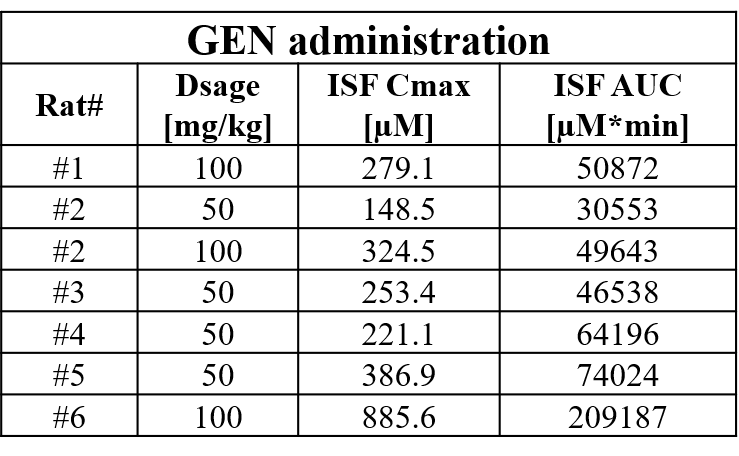
**

**
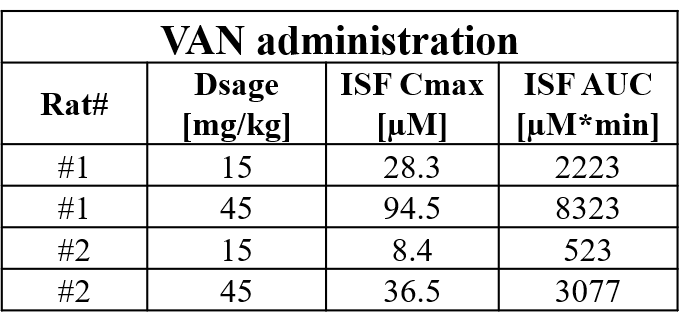
**

**Table S3.** The sequence of the GEN and VAN aptamers purchased from IDT.

| Aptamer sequence | Target | Reference paper |
| --- | --- | --- |
| /5ThioMC6-D/GGGACTTGGTTTAGGTAATGAGTCCC/3MeBlN/ | GEN | [6] |
| /5ThioMC6-D/CGAGGGTACCGCAATAGTACTTATTGTTCGCCTATTGTGGGTCGG/3MeBlN/ | VAN | [1] |

**References**

[1] P. Dauphin-Ducharme *et al.*, “Electrochemical Aptamer-Based Sensors for Improved Therapeutic Drug Monitoring and High-Precision, Feedback-Controlled Drug Delivery,” *ACS Sens*, vol. 4, no. 10, pp. 2832–2837, 2019, doi: 10.1021/acssensors.9b01616.

[2] N. Arroyo-Currás, J. Somerson, P. A. Vieira, K. L. Ploense, T. E. Kippin, and K. W. Plaxco, “Real-time measurement of small molecules directly in awake, ambulatory animals,” *Proc Natl Acad Sci U S A*, vol. 114, no. 4, pp. 645–650, 2017, doi: 10.1073/pnas.1613458114.

[3] A. Idili *et al.*, “Seconds-resolved pharmacokinetic measurements of the chemotherapeutic irinotecan: In situ in the living body,” *Chemical science (Cambridge)*, vol. 10, no. 35, pp. 8164–8170, 2019, doi: 10.1039/c9sc01495k.

[4] Y. Wu *et al.*, “Microneedle Aptamer-Based Sensors for Continuous, Real-Time Therapeutic Drug Monitoring,” *Analytical chemistry (Washington)*, vol. 94, no. 23, pp. 8335–8345, 2022, doi: 10.1021/acs.analchem.2c00829.

[5] S. Lin *et al.*, “Wearable microneedle-based electrochemical aptamer biosensing for precision dosing of drugs with narrow therapeutic windows,” *Sci Adv*, vol. 8, no. 38, pp. 1–14, 2022, doi: 10.1126/sciadv.abq4539.

[6] H. Li, P. Dauphin-Ducharme, G. Ortega, and K. W. Plaxco, “Calibration-Free Electrochemical Biosensors Supporting Accurate Molecular Measurements Directly in Undiluted Whole Blood,” *J Am Chem Soc*, vol. 139, no. 32, pp. 11207–11213, 2017, doi: 10.1021/jacs.7b05412.
